# *Yersinia pestis* and *Yersinia pseudotuberculosis* infection: a regulatory RNA perspective

**DOI:** 10.3389/fmicb.2015.00956

**Published:** 2015-09-17

**Authors:** Luary C. Martínez-Chavarría, Viveka Vadyvaloo

**Affiliations:** ^1^Departamento de Patología, Facultad de Medicina Veterinaria y Zootecnia, Universidad Nacional Autónoma de México, MéxicoMexico; ^2^Paul G. Allen School for Global Animal Health, Washington State University, Pullman, WAUSA

**Keywords:** post-transcriptional regulation, RNA-binding proteins, sRNAs, riboswitch, Hfq

## Abstract

*Yersinia pestis*, responsible for causing fulminant plague, has evolved clonally from the enteric pathogen, *Y. pseudotuberculosis*, which in contrast, causes a relatively benign enteric illness. An ~97% nucleotide identity over 75% of their shared protein coding genes is maintained between these two pathogens, leaving much conjecture regarding the molecular determinants responsible for producing these vastly different disease etiologies, host preferences and transmission routes. One idea is that coordinated production of distinct factors required for host adaptation and virulence in response to specific environmental cues could contribute to the distinct pathogenicity distinguishing these two species. Small non-coding RNAs that direct posttranscriptional regulation have recently been identified as key molecules that may provide such timeous expression of appropriate disease enabling factors. Here the burgeoning field of small non-coding regulatory RNAs in *Yersinia* pathogenesis is reviewed from the viewpoint of adaptive colonization, virulence and divergent evolution of these pathogens.

## Introduction

### *Yersinia pestis* and *Yersinia pseudotuberculosis*: so Similar Yet so Different

The Gram-negative genus *Yersinia* comprises 17 different species, but only three have been shown to be virulent to humans and animals: *Yersinia enterocolitica*, *Y. pseudotuberculosis* and *Y. pestis*. Although both *Y. pseudotuberculosis* and *Y. enterocolitica* are enteric pathogens, they are much less closely related at a DNA level than *Y. pestis* is with *Y. pseudotuberculosis*. In fact, *Y. pseudotuberculosis* diverged from *Y. enterocolitica* between 41 and 186 million years ago, while *Y. pestis* diverged from *Y. pseudotuberculosis* within the last 1500–20 000 years, which implies that these two species are more closely genetically related (Achtman et al., 1999).

Interestingly, however, *Y. pestis* and *Y. pseudotuberculosis* produce very different clinical diseases in their hosts and they use both a radically different mechanism of transmission as well as arsenal of virulence factors. While the latter is an enteropathogen which causes self-limiting food-borne enteric diseases that rarely lead to death, the former is a zoonotic pathogen that causes plague, a vector-borne disease transmitted by fleas and one of the most deadly diseases.

*Yersinia pseudotuberculosis* can be hosted in various animal reservoirs, e.g.: dogs, cats, cattle, horses, rabbits, deer, turkey, ducks, and many others, as well as in soil, plants insects and amoeba in the environment. *Y. pseudotuberculosis* infection generally occurs after ingestion of contaminated food or water, after which it colonizes the gastrointestinal tract, first at the Peyer’s patches of the distal small intestine and then it disseminates to the liver and spleen either directly or via the mesenteric lymph nodes. Infections of *Y. pseudotuberculosis* in humans usually lead to gastroenteritis, characterized by a self-limiting mesenteric lymphadenitis and diarrhea. Generally antibiotics are not even required; however, in people with underlying chronic liver ailments or that are immunocompromised, infection has been associated with septic complications and they can develop severe and potentially fatal systemic infections. Enteric *Yersinia* can spend significant portions of their lives outside the mammalian host in soil and water environments and within free-living amoeba (Lambrecht et al., 2013; Santos-Montanez et al., 2015) with varied sources of nutrients, and therefore they retain metabolic capabilities that have been lost in *Y. pestis*.

On the other hand, plague is acquired mainly by the bite of an infected flea, but also by contact with infected tissues or inhalation of respiratory droplets or aerosols. Fleas acquire *Y. pestis* from the blood of a highly bacteremic host and the bacteria multiply within the flea proventriculus and midgut forming a thick coherent biofilm that occludes, and eventually blocks, the foregut proventriculus and esophagus (Hinnebusch et al., 1996; Vadyvaloo et al., 2010). Transmission of *Y. pestis* from the fleas occurs mainly through the proventricular blockage regurgitation mechanism after a period of extrinsic incubation during which the bacteria have to adapt to the flea gut environment. This blockage impedes fresh bloodmeal ingestion and facilitates reflux from the midgut to the flea feeding mouthparts, thus causing the flea to starve. The “blocked” fleas will often regurgitate a mixture of blood and *Y. pestis-*bearing blockage material back into the flea bite site (Jarrett et al., 2004). After flea bite, *Y. pestis* transiently colonizes the dermis of the host, interacts mainly with macrophages which are permissive to its survival and replication (Pujol and Bliska, 2003; Shannon et al., 2015), and then rapidly migrates to the regional lymph node. The bacteria multiply to high numbers in the lymph node causing necrosis and inflammation that characterize bubo formation (Sebbane et al., 2005). From the bubo, hematogenous spread of the bacteria occurs to deeper organs like the spleen and liver causing septicemia. It is via this route that *Y. pestis* can also reach the lungs, to cause secondary pneumonic plague. Person-to-person spread of primary pneumonic plague can then ensue by direct inhalation of infectious droplets or aerosols through coughing.

Primary pneumonic plague is the deadliest form of plague because of its rapid progression to death in 3–4 days (Lathem et al., 2005; Bubeck et al., 2007; Price et al., 2012). Once the bacteria enter the lung tissues via the respiratory route they multiply at an accelerated rate within the first 24–36 h. This time period of the infection is characterized by absence of histopathological changes in lung tissue architecture, and anti-inflammatory molecules, thus marking an anti-inflammatory phase (Lathem et al., 2005; Bubeck et al., 2007; Price et al., 2012). Beyond the 36 h, the bacteria continue to multiply to up to 10^10^ cfus, within 3 days, at which time the bacteria disseminate from the lungs to peripheral tissues like the spleen and continue to multiply there. This latter stage of infection is characterized by raised levels of cytokines and chemokines and leads to a purulent multifocal severe exudative bronchopneumonia (Lathem et al., 2005) and death. Plague infection can be fatal without antibiotic treatment early during infection.

Surprisingly *Y. pseudotuberculosis* and *Y. pestis* differ radically in their pathogenesis despite sharing >97% identity in 75% of their chromosomal genes (Chain et al., 2004). This fact has arisen much interest in elucidating what factors are responsible for such virulence differences (Achtman et al., 2004; Pouillot et al., 2008). There is, however, one critical plasmid-encoded virulence factor that is conserved between *Y. pestis* and *Y. pseudotuberculosis*, and this is the Yop-Ysc Type three secretion system (T3SS; Cornelis et al., 1998). Most *ysc* genes encode proteins that form an injectisome structure that is required to deliver Yop effector proteins into the cytosol of host cells, e.g., immune cells. Carefully orchestrated synthesis of the T3SS Yop effector proteins is required during infection to mediate virulence. This enables the bacteria to subvert host immune function.

At the DNA level the differences between these two strains is being revealed by whole-genome sequencing based phylogenetic studies, which shows that genomes of *Y. pestis* isolates are constantly changing, acquiring and losing genetic elements and undergoing genomic rearrangements when compared with its ancestor *Y. pseudotuberculosis* (Achtman et al., 1999; Morelli et al., 2010). Some of these gene content changes between *Y. pestis* and *Y. pseudotuberculosis* have been able to explain the differences in host colonization and virulence of these pathogens (Chouikha and Hinnebusch, 2014; Sun et al., 2014; Zimbler et al., 2015). Regulatory changes and constant development of new regulatory networks are, however, also well-established ways of evolution of virulence characteristics among pathogens.

Transcriptional regulation is perhaps the most well studied form of controlling gene expression and several global transcriptional regulators play major roles in regulating virulence/pathogenesis in the yersiniae, including Crp (Zhan et al., 2008; Heroven et al., 2012b; Lathem et al., 2014), RovA (Cathelyn et al., 2006; Heroven and Dersch, 2006) and RovM (Heroven and Dersch, 2006). Few comparative studies to understand evolution of the regulons of these transcriptional factors have been undertaken in *Y. pestis* and *Y. pseudotuberculosis*. Beyond transcriptional regulation, post-transcriptional regulation which provides a powerful way for the bacteria to rapidly fine tune gene expression to the needs of the cell, at a more localized level, has recently been appreciated in *Yersinia* species.

### Small Non-Coding RNAs (sRNAs): a Mechanism of Post-Transcriptional Regulation

A major manner in which post-transcriptional regulation can be accomplished is through sRNA regulation. These are small molecules of RNA that are not translated into proteins (Gottesman and Storz, 2011). In general these molecules carry out their regulatory function by base-pairing to a limited complementary sequence (6–8 contiguous base-pairs) in the mRNAs of their cognate target gene. This interaction leads to modification of mRNA translation or stability or both, thereby influencing the target gene expression and protein activity (Narberhaus and Vogel, 2009; Waters and Storz, 2009). Small RNAs are commonly known to repress gene expression as the base-pairing sequester mRNA ribosome binding sites, resulting in translational repression and accelerated transcript degradation. However, it is emerging that they can act as translational activators and mRNA stabilizers, for which the underlying mechanisms differ considerably from repression. Translational activation occurs through interactions of sRNAs with the 5′ untranslated region (UTR), the coding sequence, or the 3′-UTR of the target mRNAs (Papenfort and Vanderpool, 2015).

Post-transcriptional regulation mediated through sRNA molecules can currently be characterized into three categories (Waters and Storz, 2009; Oliva et al., 2015): (1) Trans-encoded sRNAs: small RNAs that are distally located from, and interact through limited complementarity with their target mRNAs. These sRNAs usually bind to the Shine-Dalgarno (SD) sequence thereby occluding the ribosome-binding site (RBS), or bind to the coding region of the mRNA. Both base-pairing interactions result in inhibition of translation, and can be coupled with enhanced RNAse activity that facilitates increased rate of mRNA cleavage and degradation. The loose base-pairing interaction between the *trans*-encoded sRNA and target mRNA is often stabilized by an RNA chaperone protein, Hfq. (2) *Cis*-acting sRNAs are a second type of sRNA that is transcribed from the antisense strand of its target mRNA. Being usually encoded in the 5′-UTR region of the mRNA, it mediates its interaction by forming a duplex that contorts into a secondary structure which interferes with ribosome binding or mRNA stability. Besides the sRNA-mRNA interaction, sRNAs can directly interact with regulatory proteins to interfere with their function. This is best exemplified by the interaction of the CsrB and CsrC sRNAs with the global regulator protein CsrA which is a mechanism described for *Y. pseudotuberculosis* (Oliva et al., 2015). This will be discussed in greater detail below. (3) An alternate mechanism of post-transcriptional regulation that results in activation or repression of translation, can be achieved by conformational alterations of complex RNA structures that occurs via binding of small metabolites/cofactors (called riboswitches; Oliva et al., 2015) or thermo-modulation (called thermosensors; Krajewski and Narberhaus, 2014).

With regards to post-transcriptional regulation in the *Yersinia* species, there are several studies that have identified the arsenal of sRNAs expressed by *Y. pseudotuberculosis* and *Y. pestis* under different growth conditions. This has revealed both similarities and unexpected differences, not only limited to the presence or absence of sRNAs genes, but also related to the spatial and temporal expression patterns, and dependence on RNA binding proteins. In this review our aim is to take stock of our current understanding of the role of sRNA-mediated post-transcriptional regulation in these pathogens, and how this may have influenced the unique disease manifestations that define each species.

## sRNA Identification: What We Know in *Yersinia*

### sRNA Identification

Interest in finding small RNAs (sRNAs) in bacteria has significantly increased in recent years due to their important regulatory functions. Identification of sRNAs has been undertaken in diverse pathogenic bacterial species, e.g., *Salmonella* (Sittka et al., 2008), *Vibrio cholerae* (Liu et al., 2009), Group A *Streptococcus* (Perez et al., 2009), *Helicobacter pylori* (Sharma et al., 2010), *Clostridium difficile* (Soutourina et al., 2013), *Acinetobacter baumannii* (Sharma et al., 2014), and *Porphyromonas gingivalis* (Phillips et al., 2014) amongst numerous others. The identification and characterization of these molecules in the pathogenesis of *Yersinia*, has also in the past decade been gaining importance.

Initially sRNAs were discovered only by computational analysis using gene homology with closely related bacterial species; but without experimental data, erroneous determination of gene start sites or incorrect annotations can be made. For example, Livny et al. (2006) predicted 1478 sRNAs encoded in *Y. pestis* using sRNAPredict2 which searched for intergenic regions (IGs) with a conserved sequence and adjacent Rho-independent terminator that had to be present in 3–7 related bacterial species. Recent development of high-throughput methods and more sophisticated computational algorithms has allowed rapid identification of sRNA candidates in different species. However, given their varying sizes (50–500 nucleotides [nt]) and their potential genomic locations in the 5′- or 3′-UTRs as well as in IGs, identification and validation of true sRNAs remains challenging.

Several studies have identified multiple sRNAs expressed by *Y. pestis* and *Y. pseudotuberculosis* under various *in vitro* conditions (Koo et al., 2011; Qu et al., 2012; Beauregard et al., 2013; Yan et al., 2013; Schiano et al., 2014; Nuss et al., 2015); one study included identification of sRNAs expressed *in vivo*, in infected mouse lung and spleen organ tissue in the virulence -restricted *Y. pestis* biovar (bv.) microtus strain (Yan et al., 2013). The acronym Ysr (representing ***Y****ersinia* **s**mall **R**NAs) was initially coined by the Lathem group to name identified *Yersinia* specific sRNAs (Koo et al., 2011). Two subsequent studies assigned different names to their sRNAs (Qu et al., 2012; Yan et al., 2013), but more recently the norm has been to assign a continuous number to each newly identified *Yersinia* sRNA and prefix this with ‘Ysr.’ Moving forward the ‘Ysr’ name should be maintained to achieve standardization of *Yersinia* sRNA nomenclature.

#### *Y. pseudotuberculosis* IP32953 and *Y. pestis* CO92 sRNAs

Two pioneering studies by the Lathem group used a deep sequencing approach that enabled a comprehensive identification, validation, and partial functional characterization of a global set of sRNAs in *Y. pseudotuberculosis and Y. pestis* (Koo et al., 2011; Schiano et al., 2014). The first study, used the *Y. pseudotuberculosis* IP32953 strain, and identified 150 previously unannotated Ysrs. One hundred and eighteen of these were specific to *Y. pseudotuberculosis* and *Y. pestis* and 32 were orthologs to *E. coli* and *Salmonella* Typhimurium sRNAs (Koo and Lathem, 2012). The second study was performed using the *Y. pestis* CO92 strain and this supported and extended their previous study, as they found 144 sRNAs previously identified in *Y. pseudotuberculosis* and 63 new potential sRNAs, 10 of which were further validated by Northern blot analysis (Schiano et al., 2014). Both studies were performed essentially identically which permitted direct comparisons of the data sets. Both *Y. pseudotuberculosis* and *Y. pestis* were grown at 26 and 37°C to mimic the environment/flea and mammalian host infection temperatures, respectively, and analyzed at early log, mid log, late log and stationary phases of growth in the rich medium, brain heart infusion (BHI). The total number of potential sRNAs that they identified between *Y. pestis* and *Y. pseudotuberculosis* was 216 (Koo et al., 2011; Schiano et al., 2014). Although the majority of these sRNAs are conserved between both strains, the timing of sRNA expression and functional dependence on the post-transcriptional chaperone protein Hfq was shown to differ.

In general, in *Y. pseudotuberculosis* most Ysrs start to accumulate at later time points at both temperatures. An exception is Ysr45/GcvB which is expressed highly at the start of growth after which its expression declines over time. This was also the most abundantly expressed Ysr at 26°C, while at 37°C, Ysr7/MicA and Ysr149/OmrA/B were the most highly expressed Ysrs (Koo et al., 2011).

The sRNAs examined when *Y. pestis* grows at 26°C, show mostly steady state levels over time and little dependence on Hfq, which may explain why Hfq is not essential for *Y. pestis* survival in fleas (Rempe et al., 2012). At 37°C, however, these Ysrs are expressed differently than in *Y. pseudotuberculosis*, as most show stable levels or accumulation over time that peak at late-log phase but are almost undetectable when *Y. pestis* reaches stationary phase. Additionally, all the sRNAs tested in *Y. pestis* require Hfq for their stability/expression which may explain the severe growth defect displayed by a *hfq* mutant of *Y. pestis* at 37°C relative to that in *Y. pseudotuberculosis* (Bai et al., 2010; Koo et al., 2011; Schiano et al., 2014). This suggests that subtle evolutionary differences in post-transcriptional gene regulation exist between the two pathogenic *Yersinia* species, which may influence differential temporal regulation of targets that finally contribute to the production of divergent clinical diseases.

A group of known sRNAs (MicA/Ysr7, FnrS/Ysr11, RprA/Ysr40, GcvB/Ysr45, RybB/Ysr48, MicM/Ysr145, RyhB/Ysr146.1 and Ysr146.2, GlmY/Ysr147, GlmZ/Ysr148, and OmrA/B/Ysr149) were amongst those identified in *Yersinia* that were previously annotated and functionally characterized in *E. coli* and *Salmonella*, confirming the conservation of these sRNAs in enteric bacteria. On the other hand, a significant number of the newly discovered sRNAs contained mismatches or were absent, from the genomes of *Y. enterocolitica*, *E. coli*, and *Salmonella* Typhimurium. Many of the Ysrs encoded in both *Y. pseudotuberculosis* and *Y. pestis* contained subtle single or multiple variances in sequence. However expression of six unique sRNAs (Ysr29, Ysr53, Ysr70, Ysr84, Ysr94, and Ysr118) was noted for *Y. pseudotuberculosis* IP32953 (Koo et al., 2011), and five (Ysr142, Ysr143, Ysr144, Ysr163, and Ysr185) for *Y. pestis* CO92 (Schiano et al., 2014); these sRNAs may reflect specific and unique regulatory adaptations to host environments, and disease states caused by each of the mentioned *Yersinia* species (Supplementary Table S1).

#### *Y. pestis* KIM6+ sRNAs

Another study using a deep sequencing approach in *Y. pestis* was reported by Beauregard et al. (2013), where they identified 31 sRNAs of which only 17 matched previously identified putative sRNAs. They mapped the 5′ ends of 18 and the 3′ ends of 28 sRNAs, finding that some of them overlap an annotated protein-coding gene. All of these were conserved between *Y. pestis* and *Y. pseudotuberculosis* and all but two are conserved in *Y. enterocolitica*. Only 14 were conserved in *E. coli* but several were only partially conserved, suggesting that even when sRNAs are conserved, their functions could have diverged. Some sRNAs are conserved only in the region required for base-pairing with targets identified in *E. coli* which suggests that these Ysrs share some mRNA targets besides their species-specific targets. Similar to the findings of Koo et al. (2011), a wide variety of sRNA expression patterns differed between *Y. pestis* and *Y. pseudotuberculosis* depending upon temperature and the presence of Hfq. Comparative Northern blot analysis of all 31 sRNAs in both *Y. pestis* and *Y. pseudotuberculosis* showed that most of them were constitutively expressed in both species regardless of temperature or the presence of Hfq (Supplementary Table S1).

#### *Y. pestis* biovar Microtus strain 201 sRNAs

Two other sRNA identification analyses were undertaken in the enzootic *Y. pestis* strain 201, which is avirulent in humans, but highly virulent to mice and belongs to the newly established, bv. microtus (Zhou et al., 2004; Qu et al., 2012; Yan et al., 2013). The first study used a supposed improved cDNA cloning approach to find novel sRNAs expressed in *Y. pestis* in chemically defined TMH medium in exponential and stationary growth phases, and under stressful conditions that *Y. pestis* might encounter during infection, e.g., iron starvation, Ca^2+^ deprivation and low Mg^2+^. For stressful conditions early exponential grown cultures at 26°C were transferred to 37°C. They identified a total of 43 sRNAs. Six of these were previously annotated, 25 were encoded on the antisense strand of annotated genes or non-coding RNAs, 12 were located in IGs, and 8 were not reported previously (Qu et al., 2012). However, sRNAs were not categorized under their condition of expression because even though bacteria were grown in five separate treatment conditions, the RNA isolated from each condition was pooled in equimolar ratios for the subsequent cDNA library construction. However, four sRNAs were individually detected in the five growth conditions by Northern blot analysis: Yp-sR1, Yp-sR2, Yp-sR16 and Yp-sR38 (Qu et al., 2012). Comparisons with the Rfam database (http://rfam.sanger.ac.uk), revealed identification of six homologs of known sRNAs of other enteric bacteria, including 6S RNA, SsrA, 4.5S RNA, CyaR, CopA and STnc490. Of these, 6S RNA, which is highly abundant in *E. coli* (Hindley, 1967), was found under all five tested conditions but reached maximal abundance at stationary phase. Four sRNAs that were discovered, are absent in *Y. pseudotuberculosis* and unique to the *Y. pestis* genome, of which the Yp-sR33 is specific to *Y. pestis* bv. microtus and the CO92 genomes. Unfortunately none of these were analyzed by Northern blot or RT-qPCR to further confirm their presence (Qu et al., 2012). However, Northern blot analysis verified the presence and size of six unique sRNAs not previously identified (Supplementary Table S1). Additionally, RT-qPCR analysis of expression of a collective set of 24 sRNAs shows they are highly abundant upon entry into stationary growth phase.

In the chromosome of the *Y. pestis* 201 strain, 36 identical copies and 63 highly homologous sequences of a sRNA called Yp-sR27 was found. These highly repetitive sequences possess all the features of typical transposons, except that they do not encode a transposase and are thus termed non-inserting sequences. Non-inserting sequences are present in the ancestors of *Y. pestis*, *Y. pseudotuberculosis*, and *E. coli*, indicating that they might have evolved conservatively among bacteria. The process of the replication of these sequences remains unknown.

The second study undertaken investigated sRNA transcriptome profiles of the *Y. pestis* bv. microtus strain, grown in TMH (using the same five conditions tested above), BHI and from infected mouse lungs and spleens (Yan et al., 2013). One hundred and four sRNAs were identified, 26 already annotated and 78 representing a novel set of sRNA candidates in *Y. pestis*, 62 of which were intergenic and 16 located on the antisense of annotated ORFs. Sixty two sRNAs were identified in all four conditions tested. Four known sRNAs were found to be conserved in *E. coli* and *Salmonella* species too. However, 93 out of 104 were conserved in *Y. pseudotuberculosis* and *Y. pestis* and only 7 sRNAs were specific to *Y. pestis*. Five of the *Y. pestis* specific sRNAs were located on the *Y. pestis* pMT1 and pPCP1 plasmids indicating that acquisition of sRNA may have an important function during the evolution of *Y. pestis* from *Y. pseudotuberculosis.*

Recently, several of these sRNAs (e.g., sR028, sR041, sR050, sR066, and sR070) that were adjacently located to open-reading frames were re-annotated as 5′-UTRs (Nuss et al., 2015) in a study employing comparative RNA-seq-based high nucleotide resolution transcriptomic profiling. Because sR066 was detected as a short transcript by Northern blotting (Yan et al., 2013), it is presumed that these putative sRNAs are processed forms of a premature 5′-UTR transcript.

#### *Y. pseudotuberculosis* YPIII sRNAs

A most recent study, identified sRNAs in *Y. pseudotuberculosis* YPIII and its derivative *crp* mutant. This study employed a more rigorous and comprehensive methodology than previous studies, and determined at single nucleotide resolution the global gene expression of these bacteria grown in LB to exponential and stationary phases at 25 and 37°C (Nuss et al., 2015). A total of 78 putative *trans*-encoded sRNAs, of which 42 were new annotations, and 80 putative antisense RNAs were identified, making this the largest account of antisense sRNAs in the *Yersinia*. Nineteen antisense sRNAs were located on the virulence plasmid supporting the prevailing idea that finely controlled synchronous expression of the T3SS delivery and effector system is required during infection. A comprehensive set of 36 of these sRNAs that comprised 12 known and conserved sRNAs in the *Enterobacteriaceae*, two non-validated sRNAs (Ysr100 and Ysr103), a previously validated sRNA (Ysr164), 11 new sRNAs and 10 antisense sRNAs were confirmed by Northern blot. Most *trans*-encoded sRNAs were largely temperature and growth phase responsive in keeping with studies described above and reflect the transition of the bacteria between heterothermic environments during transmission. This study has to date advanced identification of post-transcriptional regulation involving sRNAs in *Yersinia* and allowed clear categorization of sRNAs into trans-encoded or antisense sRNAs, or as 5′-UTR regulatory elements.

#### Factors Influencing sRNA Identification

Taking the five studies together, we determine the number of sRNAs identified in *Y. pestis* and *Y. pseudotuberculosis* thus far to be ~354, of which about 105 have been alternately validated by Northern or RT-qPCR (Supplementary Table S1). Of all these identified Ysrs, 13 have been tested in mice, but only three (Ysr29, Ysr35, and tmRNA/ssrA) have been shown to be attenuated (Supplementary Table S1).

**FIGURE 1:**
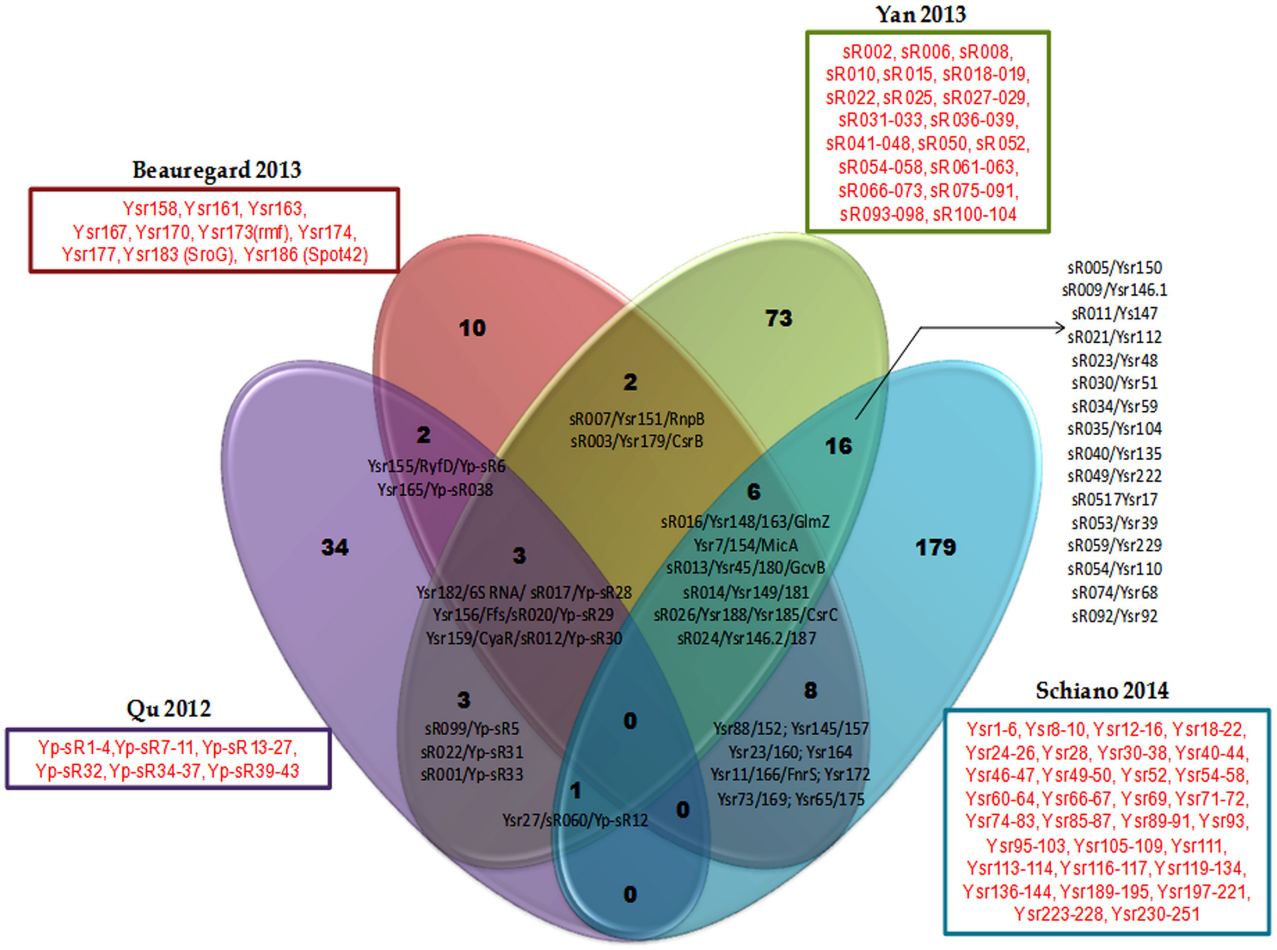
**Common (black font) and exclusively expressed (red font) sRNA repertoires are found in *Yersinia pestis* strains of genetically distinct backgrounds in four separate studies using different growth conditions.** Common sRNAs are represented by all their annotated names (these are separated by a slash) from different *Yersinia* and *E. coli* studies. Exclusively expressed consecutive sRNAs are not separately named, instead an encompassing dash indicates that they compose the list. Those non-consecutive exclusively expressed sRNAs are named singly and separated by commas from other sRNAs. Each study identifying a sRNA repertoire is represented in the Venn diagram by a different color and denoted according to the first author and year of publication of the study.

While there exists some overlap in the sRNA sets discovered in each of these studies, there are a large number of potential sRNAs that are unique to each study as exemplified in a comparison of *Y. pestis* sRNAs identified in the four studies above (**Figure 1**, Supplementary Table S2). The variable sRNA expression profiles in the studies are likely a consequence of several experimental factors: (1) specific experimental culture conditions, e.g., medium composition, temperature, phase of growth, *in vivo* vs. *in vitro*, (2) species and strains, e.g., *Y. pestis* vs. *Y. pseudotuberculosis* or *Y. pestis* CO92 vs. *Y. pestis* KIM6+ (3) methodologies used, e.g., deep sequencing vs. cDNA cloning, (4) bioinformatics analysis and pipelines applied to data, e.g., expression cut-off thresholds. However other intrinsic factors could also impact variable expression of sRNAs. Take for instance a scenario in which *Y. pestis* and *Y. pseudotuberculosis* encode the same sRNA but that this sRNAs controls different mRNAs targets in the two species, which in turn are additionally differentially regulated by distinct global regulators or growth conditions. In this case, the expression of the sRNA will vary according to the availability of its targets, as in the absence of the mRNA target the sRNA is subject to destabilization and rapid degradation. Indeed, it has been demonstrated that conserved sRNAs between *Y. pseudotuberculosis* and *Y. pestis* can differ in stability and/or expression (Koo et al., 2011; Beauregard et al., 2013). Furthermore, many of the sRNAs that are encoded and expressed by both species contain single nucleotide variations, mismatches, insertions or deletions which could alter the RNA secondary structure and result in distinct interactions with target mRNAs between the species. Some other sRNAs are duplicated, like RyhB (Ysr146.1 and Ysr146.2) in *Y. pseudotuberculosis*, which is regulated by the iron level. It would be interesting to analyze if those duplicated sRNAs have different roles in response to different environmental conditions during the infection and accordingly, if they control the expression of different sets of genes. An added complication can occur during infection if only a fraction of the cell population expresses a particular sRNA. For example, it has been shown that only a small fraction of either *Y. pseudotuberculosis* or *Y. pestis* bacterial populations express the sRNAs Ysr35 or Ysp8, at low copy numbers of 0 to 10 transcripts per cell. Such low quantities can be difficult to detect, limiting identification of such sRNAs (Shepherd et al., 2013). Certainly these differences in sRNA expression between the two pathogens are responsible for some of the divergent clinical disease outcomes.

Although, alternate validation of sRNA expression using techniques such as Northern blotting to avoid false positive identification of sRNAs is important, one is cautioned that such analysis can be tenuous especially for sRNAs expressed at low abundance. Additionally sRNAs may be expressed under conditions not examined thus far, such as upon host-cell contact, inside the cells, during animal infection or even inside the flea in case of *Y. pestis*.

Overall, studies by the Lathem group (Koo et al., 2011; Schiano et al., 2014) proved highly informative because their identification of sRNAs was accompanied by further verification of their expression by Northern analysis, investigation of sRNA Hfq-dependency, identification of cognate mRNAs targets and functional analysis of newly identified sRNAs that revealed their roles in virulence. On the other hand the Nuss et al. (2015) study has used improved techniques to identify and categorize sRNAs, as well, they managed to pinpoint Crp as another major sRNA regulator and verify its direct targeting of several sRNAs.

## Functional Characterization of sRNAs in *Yersinia*

Currently >350 Ysrs have been identified but the functional roles that most of these molecules play are yet to be discovered. The functional roles for known *Yersinia* sRNAs conserved in *E. coli* and other related pathogens, that have been subject to thorough investigation of their function, mechanism of action, mRNA targets and conditions of expression are usually inferred. However, the physiological and virulence-inducing processes have become the focus of functional studies on Ysrs, and such roles have been established for a small number of these Ysrs thus far. In this section we will discuss some of these findings.

### Virulence

#### SsrA/sR022: a Vaccine Candidate for Pneumonic Plague?

Small stable RNA A (SsrA RNA), also known as tmRNA or 10S, and small protein B (SmpB) are components of a unique bacterial translational control system (Karzai et al., 2000). This system is important for maintaining cellular homeostasis and bacterial survival in adverse conditions, and as such may be advantageous for efficient response to adverse infection conditions. The SsrA-SmpB system helps maintain the bacterial translational machinery in a fully operational state by dealing with ribosomes that are stalled on defective mRNAs that lack stop codons (Karzai et al., 2000; Dulebohn et al., 2007; Keiler, 2008). SsrA RNA functions both as a tRNA and mRNA. Whereas SmpB is essential for recognition and delivery of SsrA to target stalled ribosomes, as it binds specifically to the tRNA-like domain of SsrA and thus stabilizes SsrA tertiary structure (Karzai et al., 2000; Barends et al., 2001).

The role of SsrA was first evaluated in *Y. pseudotuberculosis* but among deep sequence analyses that searched for new *Yersinia* sRNAs, it was identified in the *Y. pestis* bv. microtus strain and referred to as sR022 or Yp-sR31 (Yan et al., 2013). In *Y. pseudotuberculosis*, expression of VirF, a key TTSS transcriptional activator, and secretion of Yops (YopB, YopD, YopM, LcrH, and LcrV), are reduced in the *smpB-ssrA* mutant. Furthermore this mutant exhibits delayed host cell cytotoxicity and is more sensitive to oxidative and nitrosative stresses, low pH, and sublethal concentrations of translation-specific antibiotics, and is non-motile (Okan et al., 2006, 2010). Consequently, the *smpB-ssrA* mutant strain is avirulent to mice via the orogastric route, as well as defective in survival and replication in macrophages (Okan et al., 2006). It also showed partial protection when challenged with a lethal dose of *Y. pestis*.

In *Y. pestis*, a *smpB-ssrA* mutant is severely attenuated in a mouse model of infection via both the intranasal and intravenous routes. This mutant exhibits a slower growth rate at 37°C (Okan et al., 2010). In agreement, the transcriptional level of SsrA is higher at 37°C than that at 26°C in *Y. pestis* bv. microtus (Qu et al., 2012), which may help *Y. pestis* adapt to natural temperature alterations during its transmission from fleas to mammals. Most significantly, it has been demonstrated that intranasal vaccination of mice with the *ssrA* mutant induced a strong IgG antibody response, and vaccinated animals were well protected against pulmonary *Y. pestis* infection (Okan et al., 2010). Taken together, these characteristics present this strain as a favorable candidate for a live attenuated cell-based vaccine against pneumonic plague.

#### Ysr141

Ysr141 is present in both *Y. pseudotuberculosis* and *Y. pestis* as identified by sequencing analysis. Its genomic location is mapped to the T3SS-carrying plasmid, pCD1 and it is encoded on the opposite strand within the IG between *yopH* and the gene YPCD1.68c (Koo et al., 2011; Schiano et al., 2014).

Ysr141 is an unstable sRNA which stimulates production of T3SS-associated effector proteins (YopE, YscF, YopK, and LcrF) and regulates *yopJ*, posttranscriptionally, by base-pairing to the 5′-UTR of *yopJ* (Schiano et al., 2014). It has been suggested that Ysr141 may be a mechanism to regulate the T3SS, conserved among the pathogenic *Yersinia* species and it may link environmental cues to the modulation of the T3SS in such species. An investigation into the environmental and temporal cues that trigger expression of Ysr141 and its activation of the T3SS could potentially provide further insight into the differing clinical disease manifestations in *Y. pseudotuberculosis* and *Y. pestis*.

#### Ysr35

This sRNA was identified from deep sequence analysis (Koo et al., 2011; Schiano et al., 2014). It has been suggested that this sRNA could be required for *Yersinia* adaptation to the host because a Ysr35 mutant showed significantly compromised survival in a mouse model in both *Y. pseudotuberculosis* and *Y. pestis* (Koo et al., 2011). The expression of Ysr35 has been evaluated by measurement of gene expression in *Y. pseudotuberculosis* using single-molecule fluorescence hybridization (smFISH) and its expression was demonstrated to increase upon a temperature upshift from 25 to 37°C, further supporting its importance in pathogenesis (Shepherd et al., 2013).

#### CsrB and CsrC

These two sRNAs were first described in *Y. pseudotuberculosis* where they were found to be essential for the initial phase of the infection in *Yersinia* species (Heroven et al., 2008, 2012a). CsrC has also been identified by deep sequencing analysis in *Y. pseudotuberculosis* (called Ysr188, Koo et al., 2011), in *Y. pestis* strain 201 (called sR026, Yan et al., 2013), and in *Y. pestis* KIM6+ (called Ysr185, Beauregard et al., 2013), whereas CsrB has been only identified in *Y. pestis* by Beauregard et al. (2013) and Yan et al. (2013) and called Ysr179 and sR003, respectively. These sRNAs has been widely studied in many bacteria as they are part of the Csr system, along with the RNA binding protein CsrA, as such they will be described in the next section below.

### Metabolism

#### RyhB/Ysr48: a Key Regulator of Bacterial Iron Metabolism

The RyhB/Ysr48 sRNA, conserved in *E. coli* and in other *Enterobacteriaceae*, is involved in the post-transcriptional regulation of numerous genes during iron depletion (Masse and Gottesman, 2002; Masse et al., 2003, 2005; Murphy and Payne, 2007; Alice et al., 2008). It is also involved in bacterial growth, biofilm formation, chemotaxis, acid resistance and intracellular growth (Mey et al., 2005; Bollinger and Kallio, 2007; Murphy and Payne, 2007; Boughammoura et al., 2008; Guillemet and Moreau, 2008).

*Yersinia pestis* encodes two RyhB homologs, RyhB1 and RyhB2, that are located some distance from each other on the chromosome. RyhB1 and RyhB2 share a sequence with *E. coli* and *Salmonella* Typhi that ranges from 61 to 72% (Deng et al., 2012). These sRNAs were identified in deep sequencing studies of *Y. pseudotuberculosis* (Ysr146.1 and Ysr146.2, Koo et al., 2011), virulence-restricted *Y. pestis* bv. microtus (sR009/sR024, Qu et al., 2012) and *Y. pestis* C092 (Ysr146.1/Ysr146.2, Schiano et al., 2014), whereas only RyhB2 was detected in *Y. pestis* KIM (Ysr146.2/187, Beauregard et al., 2013). Both sRNAs are induced by iron deficiency and are negatively regulated by the ferric uptake regulator, Fur (Deng et al., 2012).

The stabilization of RyhB1 and RyhB2 is differentially dependent on Hfq, with RyhB1 stabilization being mediated by Hfq, and that of RyhB2 not (Deng et al., 2012). Both sRNAs are strongly expressed in lungs of mice infected intranasally with *Y. pestis* which indicates that signals such as iron depletion may be present in the infected lungs (Deng et al., 2012). However it was shown that a *ryhb1-ryhB2* double mutant has no discernable defect in survival and dissemination in the host after intranasal inoculation, which suggests that other iron uptake systems have compensatory effects in this environment.

#### GlmY and GlmZ: Cell Wall Synthesis

These sRNAs regulate synthesis of the enzyme glucosamine-6-phosphate (GlcN6P) synthase, GlmS, in *Enterobacteriaceae*, which catalyzes formation of GlcN6P, the initial building block in the pathway that generates precursors of cell wall synthesis (Urban et al., 2007; Reichenbach et al., 2008; Urban and Vogel, 2008). The GlmY and GlmZ sRNAs are the only known direct targets of the GlrR/GlrK two component system, where GlrK is the sensor kinase that phosphorylates GlrR, which is the response regulator that directly binds to their promoters. GlrR induces *glmY* expression through activation of the σ54-promoter, when cells enter the stationary growth phase (Reichenbach et al., 2009). It has been shown that overlapping σ54- and σ70-promoters direct expression of *glmY* gene, while expression of *glmZ* is achieved from a single constitutively active σ70-promoter (Gopel et al., 2011).

Homologous sRNAs to GlmY and GlmZ have been identified in both *Y. pestis* and *Y. pseudotuberculosis* (Koo et al., 2011; Beauregard et al., 2013; Yan et al., 2013; Schiano et al., 2014). Although their role in virulence has not been evaluated, in *Y. pseudotuberculosis*, these sRNAs are transcribed from σ54-dependent promoters and are induced by the two component system GlrR/GlrK, through direct binding of GlrR to sites located upstream of their promoters (Gopel et al., 2011). In addition, it was shown that putative binding sites for the integration host factor, IHF, are present in the *glmY* and *glmZ* promoter regions, suggesting that IHF could be involved in their regulation, maybe facilitating interaction of GlrR with the σ54-RNA polymerase by binding-induced bending of critical promoter sequences, consistent with the usual role of IHF (Swinger and Rice, 2004).

#### SraG

First reported in *E. coli*, SraG located between *pnp* (PNPase) and *rpsO* (30S ribosomal protein S15), is expressed preferentially at late-logarithmic phase, and activated by heat and cold shock treatments (Argaman et al., 2001; Sridhar et al., 2009). A comparative sequence analysis with *E. coli* revealed a SraG homolog in *Yersinia* species (Sridhar et al., 2009). In *Y. pseudotuberculosis* 16 proteins were identified as potential regulatory targets of SraG (Lu et al., 2012). However, only *pnp* and YPK_1205 showed significantly different mRNA levels when a RT-PCR validation of these targets was performed. It was shown that SraG negatively regulates the YPK_1206-1205 operon post-transcriptionally by likely targeting the coding region of YPK_1206. The YPK_1206-1205 operon is present only in *Y. pseudotuberculosis* and *Y. enterocolitica* and they share 90% similarity. Unfortunately the role of this operon has not been elucidated but YPK_1206 is predicted to have roles in DNA bending, therefore SraG could be acting as a regulatory element in this process (Lu et al., 2012). As three of the 16 potential targets correspond to proteins associated with maltose metabolism, it has been suggested that SraG might also be involved in regulation of maltose metabolism. This is a classic example of a sRNA that is not conserved in *Y. pestis* but may contribute to adaptation of *Y. pseudotuberculosis* to its host, e.g., maltose is found in the human gut when it is broken down from grains like wheat.

#### GcvB/Ysr45

Initially predicted using a bioinformatics search of the *Y. pestis* genome. The *gcvB* gene is adjacent to, and divergent from *gcvA* that shares considerable sequence homology (77%) with the *E. coli gcvB* sequence (McArthur et al., 2006). Deep sequence analysis of both *Y. pestis* and *Y. pseudotuberculosis* have also identified GcvB (where it has been called Ysr45, Ysr45/180 or sR013; Koo et al., 2011; Beauregard et al., 2013; Yan et al., 2013; Schiano et al., 2014).

*Yersinia pestis gcvB* encodes two sRNAs that repress expression of *dppA* that encodes a periplasmic-binding protein component of the dipeptide transport system (McArthur et al., 2006). Deletion of the *gcvB* gene in *Y. pestis*, results in altered growth rate and colony morphology, and due to the pleiotropic nature of these effects it has been suggested that this sRNA is a global regulator of multiple downstream genes in addition to *dppA*, similar to its function in *E. coli* (Urbanowski et al., 2000; McArthur et al., 2006). These target genes remain to be identified.

In *Y. pestis*, transcription of *gcvB* is activated by the GcvA protein and repressed by the GcvR protein. A comparison of the *gcvB* regulatory regions in *Yersinia* species have shown that the putative GcvA binding sites for activation of *gcvB*, are 100% identical in all *Y. pestis* strains, and >92% identical in other *Yersinia* species, which suggests that the regulatory mechanisms of the GcvB RNAs are possibly similar in all *Yersinia* species.

#### SgrS

SgrS is an Hfq-dependent sRNA that has been widely studied in *E. coli*, where it regulates the *ptsG* mRNA, which encodes the major glucose transporter PtsG, by occluding the *ptsG* RBS leading to degradation of *ptsG* (Kawamoto et al., 2006).

This sRNA also functions as an mRNA, as it encodes the protein SgrT which is ectopically produced under glucose-phosphate metabolic stress conditions when cells are unable to appropriately metabolize phosphorylated sugars (Wadler and Vanderpool, 2007). SgrS prevents new sugar transporters from being produced under conditions where the accumulated sugar-phosphates have become toxic; in so doing it maintains continued cell growth under such conditions (Vanderpool and Gottesman, 2004; Wadler and Vanderpool, 2007).

In *Yersinia* this sRNA was found by bioinformatics analysis aimed at finding homologs of *E. coli* SgrS. The *Y. pestis* SgrS homolog is, however, truncated at the 5′ end such that it is devoid of the *sgrT* coding sequence and retains only typical sRNA base-pairing function (Horler and Vanderpool, 2009). This sRNA has been detected by deep sequencing in both *Y. pestis* and *Y. pseudotuberculosis*, where it has been called Ysr150 or sR005 (Koo et al., 2011; Yan et al., 2013; Schiano et al., 2014).

The *Y. pestis sgrS* ortholog is able to base pair with the *E. coli* K12 *ptsG* mRNA and inhibit its translation (Wadler and Vanderpool, 2007), however, it fails to inhibit growth on minimal glucose medium because the *sgrT* CDS is missing. It is still able to promote recovery of an *E. coli sgrS* mutant strain from glucose-phophate stress, which indicates it conserves the function of regulating target gene expression; the base pairing function of *E. coli* and *Y. pestis* SgrS homologs is critical for rescue from glucose-phosphate stress (Wadler and Vanderpool, 2007).

#### YsrH

YsrH is a novel *cis*-encoded sRNA identified as Yp-sR16 by Qu et al. (2012) in the avirulent *Y. pestis* bv. microtus strain. This sRNA is conserved in all *Yersinia* species and located on the opposite strand to *fabH2*, which encodes β-ketoacyl-acyl carrier protein synthase III, an enzyme essential for fatty acid biosynthesis in bacteria (Qu et al., 2012; Lu et al., 2014). YsrH is expressed in the early-exponential growth phase and this expression is maintained at the same level in later stages of growth (Lu et al., 2014). It has been reported that YsrH negatively regulates the fatty acid synthesis post-transcriptionally by specifically targeting *fabH2* mRNA transcripts for degradation and this mechanism also involves the PNPase and RNase E-associated processing pathways (Lu et al., 2014).

### Stress Response

#### Ysr29

Ysr29 was identified in reports by Koo and Schiano (Koo et al., 2011; Schiano et al., 2014) but is specific to the *Y. pseudotuberculosis* IP32953 strain. It is expressed much better at 26°C, where its expression depends on the chaperone Hfq. Ysr29 seems to negatively regulate GST and positively regulate RpsA, OmpA and GroEL at a posttranscriptional level because the absence of the sRNA does not affect their transcript levels but instead alters the levels of protein (Koo et al., 2011). GST participates in protecting cells against the damage of oxidative stress (Allocati et al., 2009), and Ysr29 repression of GST levels may prevent an aberrant response to this stress. Interestingly, reactive oxidative species produced by the flea upon infection, is a known stress that *Y. pestis* has to defend itself against during the early stages of infection (Zhou et al., 2012). Therefore, the fact that Ysr29 is not conserved in *Y. pestis* could be as a result of evolutionary selection, as loss of a negative regulator of GST, would lead to expression of GST to cope with the oxidative stress it finds in a new niche, e.g., the flea.

### *Y. pestis* Transmission from Fleas

#### HmsB: the Biofilm Regulator

Biofilm is a population of bacterial cells embedded in a self-produced exopolysaccharide (EPS) matrix. Biofilm formation results in blockage of the flea proventriculus, which facilitates *Y. pestis* transmission to new mammalian hosts (Jarrett et al., 2004; Hinnebusch and Erickson, 2008).

The second messenger 3′,5′-cyclic diguanosine monophosphate (c-di-GMP), is central to the biofilm formation as it promotes EPS production (Simm et al., 2004). In *Y. pestis* EPS biosynthesis is encoded by the *hmsHFRS* operon, whereas HmsT and HmsD are the two sole diguanylate cyclases responsible of biosynthesis of c-di-GMP, while HmsP is the sole phosphodiesterase responsible for c-di-GMP degradation (Kirillina et al., 2004; Bobrov et al., 2005, 2011; Sun et al., 2011).

HmsB is an Hfq-dependent sRNA (originally called sRNA035) identified by RNA-seq study of *Y. pestis* bv. microtus (Yan et al., 2013). This is the first sRNA to be reported as a biofilm regulator. An *hmsB* deletion reduces biofilm formation both *in vitro* and on nematodes, which is a result of the decreased production of c-di-GMP (Fang et al., 2014). These effects are linked to the fact that HmsB positively regulates *hmsCDE, hmsT, hmsHFRS* and itself, thereby enhancing biofilm production while it negatively regulates *hmsP*, encoding the biofilm-inhibiting phosphodiesterase (Fang et al., 2014). However, the exact molecular interaction of HmsB with *hmsCDE, hmsT, hmsHFRS, hmsP* and other direct targets that could be involved in this process, remain to be elucidated.

## Globally Acting RNA Binding Proteins: The Hfq and CsrA Paradigms in *Yersinia*

Post-transcriptional activation of transcripts can occur indirectly, through mechanisms that do not require direct base pairing of sRNAs with their targets. In these cases, globally acting RNA-binding proteins Hfq and CsrA often play an important role. These thoroughly studied RNA binding proteins and their roles in sRNA function in the *Yersinia* genus will be discussed below.

### Hfq: the sRNA Chaperone *par Excellence*

Hfq, a close relative of the Sm-like (Lsm) family of eukaryotic proteins, is a well-known RNA chaperone widely recognized for being required for the proper functioning of many *trans*-acting sRNAs. It promotes stable sRNA:mRNA base-paring interactions as in *trans*-acting sRNAs the contact on the target is typically short and imperfect (Jousselin et al., 2009) and it also protects the sRNA from degradation (Brennan and Link, 2007). Most sRNAs characterized to date need binding of Hfq as a RNA chaperone to stabilize the sRNA-target mRNA duplexes (Han et al., 2013). Due to its global post-transcriptional regulatory impact, *hfq* mutation typically results in pleiotropic phenotypes affecting outer membrane biogenesis, quorum sensing, virulence factor synthesis, protein secretion, virulence gene expression and general stress response pathways (Chao and Vogel, 2010; Papenfort and Vogel, 2010). Hfq binding sites have only been mapped for a limited number of transcripts; but all appear to contain a consensus AU-rich single stranded region following an RNA stem loop (Schumacher et al., 2002; Gottesman and Storz, 2011), that are able to interact with the poly-U tail of certain Rho-independent terminator containing RNAs (Otaka et al., 2011).

Hfq was first identified in yersiniae in the *Y. enterocolitica* species, as the protein Yrp which regulates production of the heat stable enterotoxin, Y-ST (Nakao et al., 1995); a subsequent global identification of *Y. enterocolitica* sRNAs has not to date been reported. The Hfq regulatory protein in *Yersinia* was first studied by Geng et al. (2009) using the enzootic *Y. pestis* strain 201. They examined the role of this sRNA chaperone in virulence and performed whole-genome transcriptomic profiling of an *hfq* mutant. Their transcriptional analyses revealed that *hfq* mutation results in dysregulation of ~243 genes in *Y. pestis*. Among the upregulated genes are those involved in macromolecule metabolism, heat shock response and virulence regulation, whereas genes belonging to classes of degradation of small molecules, or energy metabolism and oxidative stress, were downregulated. Numerous hypothetical genes were also dysregulated in the *hfq* mutant.

In general, the Hfq mutant in both *Y. pestis* and *Y. pseudotuberculosis* exhibit growth defects, at ~26 and 37°C, however, the growth defect in *Y. pestis* appears to be more severe compared to that in *Y. pseudotuberculosis* at 37°C (Bai et al., 2010; Schiano et al., 2010). Northern blot analysis of *hfq* shows that *hfq* transcript levels are substantially lower at 37°C than at 28°C in both *Y. pestis* and *Y. pseudotuberculosis* (Beauregard et al., 2013). Nevertheless Hfq is required for the full virulence of both *Y. pestis* and *Y. pseudotuberculosis* in mouse models of infection (Bai et al., 2010; Schiano et al., 2010; Koo et al., 2011). In the lower temperature flea host, an *hfq* mutant of *Y. pestis* is also compromised in its ability to form a biofilm blockage in the flea proventriculus and is outcompeted by the wild type strain during coinfection in fleas (Rempe et al., 2012). In *Y. pseudotuberculosis*, Hfq plays other important roles in sensitivity to heat, oxidative stress resistance, tolerance to long-term nutrient-limitation and antibacterial peptides, phagocytosis resistance, survival and persistence within phagocytes (Schiano et al., 2010). The effects on virulence are likely associated with the role of Hfq in post-transcriptionally regulating multiple components of the T3SS, the major virulence factors in both *Y. pseudotuberculosis* and *Y. pestis* (Schiano et al., 2010, 2014).

From the various sRNA studies in *Yersinia* it can clearly be gleaned that a complex relationship between temperature and Hfq dependence is in place for sRNA regulation between the *Yersinia* strains. So far, about 41 sRNAs have been shown to be regulated by Hfq in both *Y. pestis* and *Y. pseudotuberculosis*. For instance, the sRNA Ysr48/RyhB is Hfq-dependent in *Y. pestis* at both 37 and 26°C but requires Hfq in *Y. pseudotuberculosis* only at 37°C (Supplementary Table S1, Koo et al., 2011). The Ysr48/RyhB mutant is slightly attenuated in *Y. pseudotuberculosis* yet remains virulent in *Y. pestis* in a pneumonic plague infection model. As Hfq is 100% identical between *Y. pestis* and *Y pseudotuberculosis*, regulation by Hfq-dependent sRNAs rather than Hfq itself is what likely contributes to the differences in virulence in both species at 37°C.

Some mechanistic aspects of Hfq regulation of the sRNA HmsB, which controls biofilm formation, and RyhB which is involved in the iron metabolism, have been described in *Y. pestis*. In enzootic *Y. pestis* strain 201, the Hfq-regulated sRNA, HmsB, which controls biofilm formation, is dramatically degenerated in the absence of *hfq* (Fang et al., 2014). Yet, in the presence of *hfq* enhanced expression of HmsB occurs, which leads to increases in expression of the genes enhancing biofilm formation, which are the *hmsCDE* (Bobrov et al., 2015), *hmsT* and *hmsHFRS* genes (Hinnebusch et al., 1996); simultaneously, inhibition of expression of *hmsP* which negatively regulates biofilm formation occurs (Fang et al., 2014), resulting in increases in biofilm formation. However, this effect on biofilm formation is the opposite in the epidemic *Y. pestis* CO92 strain, where Hfq acts as a repressor of biofilm formation through inhibiting expression of *hmsT*, and *hmsHFRS* but stimulating that of *hmsP* (Bellows et al., 2012). This difference in Hfq-dependent biofilm regulation may be explained by the different conditions under which biofilm formation was tested between the two studies. However, it does not rule out that other strain-specific Hfq-regulated sRNAs involved in biofilm control may play a role here. This idea would be consistent with a role for sRNA regulation in the evolution of the distinct pathologies between ancestral and newly evolved strains because the mouse virulent *Y. pestis* strain 201 is less recently evolved than the human virulent *Y. pestis* strains (Morelli et al., 2010).

In *Y. pestis*, Hfq dependence of RyhB1 and RyhB2, the two sRNA encoded by RhyB, is different, as RyhB1 stabilization is mediated by Hfq, whereas RyhB2 does not require Hfq for stability (Deng et al., 2012). In absence of Hfq, rapid degradation of both sRNAs occurs. The ribonuclease PNPase is the main enzyme that degrades Hfq-free RyhB (Deng et al., 2014). This is different from the general mechanism of PnPase activity which usually involves PnPase formation of multi-enzyme ribonucleolytic complexes with RNase E and/or RNA helicase, RhlB, to mediate degradation of the structured RNA (Kaberdin et al., 2011; Silva et al., 2011). Further work is required to explain the mechanism by which RyhB2 maintains stability in absence of Hfq and if RyhB1 and RyhB2 have distinct targets based on their requirement for Hfq.

In spite of the well accepted role of Hfq in *Yersinia* virulence, not all Hfq-dependent sRNAs are important for virulence. For instance, 50 and 10% of mice survived after intragastric infection by the Hfq-dependent sRNAs, Ysr29 and Ysr48/RybB mutants, respectively (Koo et al., 2011), whereas 50% of mice survived after inoculation with the Hfq-independent Ysr35 sRNA mutant. Further, in *Y. pestis* neither the Hfq-independent Ysr23 nor the Hfq-dependent Ysr48 affect the ability of *Y. pestis* to cause disease and death in a pneumonic plague infection model (Koo et al., 2011). These data indicate that not all the sRNAs that depend on Hfq, play a direct role in virulence. Even for those sRNAs whose deletion resulted in a decrease of virulence, the attenuation was not as dramatic as the loss of virulence displayed by mutants lacking Hfq. Multiple Hfq-dependent sRNAs contribute to this Hfq virulence phenotype and an approach in which multiple sRNAs are deleted may be worthwhile in producing a phenotype resembling an *hfq* mutant.

The fact that the overexpression of *hfq* causes substantial decreases of some sRNAs even when the deletion of *hfq* has no substantial effect on sRNA levels suggests that Hfq expression levels itself need to be finely controlled to avoid aberrant effects. Hfq-dependence for the same sRNA can vary between bacteria. For example, MicA and GcvB whose expression/stability is known to rely on Hfq in *E. coli* and *Salmonella*, are Hfq-independent in *Y. pseudotuberculosis* (Koo et al., 2011).

The distinct expression patterns of conserved sRNAs between *Y. pestis* and *Y. pseudotuberculosis* could be what influences their different pathologies. However, alignments between the sRNAs have shown that almost all of them share >95% identity and Hfq is 100% identical between these strains. Thus the difference in expression patterns may be due to the variance in abundance of target mRNAs, as their absence or abundance would influence base-pairing interactions which would determine stability and degradation of the cognate sRNAs. This in turn, may affect the amounts of available Hfq. At the same time, the abundance of the target mRNAs is subject to first order transcriptional regulation and different environmental conditions that also influence sRNA expression.

Hfq contributes to the regulation of the global transcriptional regulator Crp in a post-transcriptional manner in *Y. pestis* at mammalian physiological temperatures (Lathem et al., 2014); but not in *Y. pseudotuberculosis* (Nuss et al., 2015). Both *crp* and *hfq* mutants of both *Y. pestis* (Bai et al., 2010; Qu et al., 2013; Lathem et al., 2014) and *Y. pseudotuberculosis* (Schiano et al., 2010; Heroven et al., 2012b) are attenuated during mouse infection. In *Y. pestis*, attenuation of the *hfq* mutant is partially attributable to Crp (Lathem et al., 2014). These findings imply that disparate Hfq-dependent sRNA regulation, factors into the different diseases between *Y. pestis* and *Y. pseudotuberculosis*.

### CsrA Control is Mediated by sRNAs, CsrC and CsrB

The global translational regulator CsrA has been discovered and studied in many bacteria and some species encode multiple paralogous regulators (Lapouge et al., 2008). This protein typically binds to GGA-rich elements in RNAs. CsrA binding to the SD sequence of bacterial mRNAs results in reduced translation and subsequent mRNA decay. Although it acts primarily as a repressor, in few cases CsrA also activates target mRNA translation (Patterson-Fortin et al., 2013; Yakhnin et al., 2013). CsrA is a major component of the Csr system along with the sRNAs CsrB and CsrC (Babitzke and Romeo, 2007). These sRNAs contain multiple binding sites for CsrA and can therefore bind and titrate CsrA away from its repressed mRNA targets – amounting to indirect activation of these mRNA targets. As a consequence of sequestration, the usual target mRNAs of the CsrA proteins are mostly upregulated (Liu et al., 1997; Weilbacher et al., 2003; Babitzke and Romeo, 2007; Lapouge et al., 2008; Duss et al., 2014). CsrB and CsrC are often found in multiple copies and are present in many bacteria where they can function redundantly.

The Csr system was identified during a search for regulators that influence the expression of the global virulence transcriptional factor RovA in *Y. pseudotuberculosis*, which activates the primary cell entry factor invasion, InvA (Nagel et al., 2001; Heroven et al., 2004; Tran et al., 2005). There are no studies to date reporting the effect of the Csr system in *Y. pestis* therefore our knowledge of CsrA is based on studies in *Y. pseudotuberculosis*. It has been elucidated that CsrA induces the expression of the LysR regulator RovM, which in turn represses *rovA*, thus CsrA represses RovA synthesis indirectly through control of RovM. CsrB and CsrC titrate and sequester CsrA which leads to the activation of *rovA* (Heroven et al., 2008). In agreement, overexpression of CsrB and CsrC induces *rovA* (Heroven et al., 2008); however, neither a *csrC* or *csrB* single mutant affects expression of *rovA*, suggesting that these sRNAs are redundant in the manner in which they regulate *rovA*. Their function in other pathogens is the same, where it has been demonstrated that the loss of CsrB causes a compensatory increase in CsrC levels and vice versa (Weilbacher et al., 2003; Fortune et al., 2006).

The global influence of the Csr system in *Yersinia* is reflected by many different physiological changes for which it is responsible. A *csrA* mutant in *Y. pseudotuberculosis* has a growth defect whereas CsrA overexpression alters cell morphology (Heroven et al., 2008, 2012a); it inhibits glycogen synthesis and about 20% of the CsrA-dependent genes are involved in metabolism (Heroven et al., 2012a). On the other hand, CsrA positively controls motility in *Y. pseudotuberculosis*. It induces flagella biosynthesis by binding directly to the transcript of *flhDC*, which encodes the master regulator of flagellum biosynthesis (Heroven et al., 2008). Absence of CsrA also affects the expression of genes involved in resistance to stress (Heroven et al., 2008). Similar to what has been reported in other microorganisms, intracellular levels of CsrA have to be tightly regulated, as both the loss and the overexpression of CsrA negatively affect invasion to cells (Heroven et al., 2008).

Orthologs of *csrB* and *csrC* genes as well as related Csr (Rsm)-type RNA genes in other bacteria, are activated by the two-component signal transduction system BarA/UvrY which regulates the expression of genes associated with virulence, secondary metabolism, motility, exoenzyme production, quorum sensing or biofilm formation (Goodier and Ahmer, 2001; Lapouge et al., 2008). In *Y. pseudotuberculosis* this scenario seems to be similar only for *csrB* expression, which is positively induced by UvrY (Heroven et al., 2008). However, only when CsrB is present, UvrY seems to repress the expression of CsrC (Heroven et al., 2008).

Recently it has been demonstrated that CsrC, but not CsrB, is regulated by the global regulator PhoP. PhoP induces the expression of CsrC from two different promoters by directly binding to two distinct sites located within the *csrC* regulatory region (Nuss et al., 2014).

Expression of CsrB and CsrC is medium dependent; *csrC* is mostly detected during growth in nutrient rich media, where *csrB* expression is very low, whereas only very low levels of CsrC are observed in minimal medium (Nagel et al., 2001; Heroven et al., 2008). Therefore the CsrA-mediated control of the RovM-RovA-InvA virulence cascade is strongly affected by changes in carbon source availability through alterations of the Csr RNAs, in particular CsrC (Nagel et al., 2001; Heroven and Dersch, 2006; Heroven et al., 2008). This links the virulence CsrABC-RovM-RovA-InvA cascade with the cAMP receptor protein Crp, a crucial global regulator that controls the transcription of multiple genes and operons in bacteria by catabolic repression in response to the glucose supply (Saier, 1998). Crp regulates the synthesis of both Csr RNAs in an opposite manner, as it activates CsrC and represses CsrB transcription (Heroven et al., 2012b). More details of role of Crp in *Yersinia* are given below.

## Crp Commands a sRNA Repertoire

More recently it is becoming apparent that the global transcriptional regulator cyclic AMP (cAMP) receptor protein, Crp, has an important role in regulating sRNAs in *Y. pseudotuberculosis* and *Y. pestis* (Lathem et al., 2014; Nuss et al., 2015). Within the *Enterobacteriaceae* family, Crp is a well-studied first order transcriptional regulator that regulates numerous target genes and operons in response to carbon sugar availability (Zhan et al., 2008). Transcriptional regulation by Crp is exacted following activation of the Crp protein by cAMP and once the cAMP-Crp complex binds to a symmetrical recognition site (TGTGA-N_6_-TCACA) in the promoter region of target genes and operons.

Importantly, mutants in the *crp* gene of *Y. pseudotuberculosis* (Heroven et al., 2012b) and *Y. pestis* (Zhan et al., 2008; Lathem et al., 2014) are attenuated in virulence. The discerning feature of the attenuated phenotype exhibited by a *crp* mutant is the inability of the bacteria to disseminate from early infection sites to deeper tissues (Zhan et al., 2008; Heroven et al., 2012b). Although the *crp Y. pestis* attenuation mainly results from decreases in expression of genes encoding the major virulence factors like Pla protease and the T3SS and associated Yop effector proteins, alterations in stress adaptation and numerous metabolic genes notably contribute to this phenotype (Zhan et al., 2008, 2009). Similarly, in *Y. pseudotuberculosis* a large proportion of genes involved in primary metabolism, stress and virulence were dysregulated in a *Y. pseudotuberculosis crp* mutant (Nuss et al., 2015). Alongside this, extensive metabolome and fluxome changes to central carbon metabolism related to the pyruvate-tricarboxylic acid cycle were noted in a *crp* mutant of *Y. pseudotuberculosis* (Bucker et al., 2014). Collectively, this data emphasized that Crp plays a role in linking nutritional status and virulence.

Expression of Crp appears to be increased at stationary phase in *Y. pseudotuberculosis* and this was demonstrated to be a function of post-transcriptional control. In *Y. pestis*, Hfq is able to post-transcriptionally control expression of Crp at mammalian host temperature through interaction with a 79 nt long 5′-UTR of Crp (Lathem et al., 2014), however, expression of Crp in *Y. pseudotuberculosis* was maintained independent of Hfq at both 37°C and 26°C (Nuss et al., 2015). In addition, the 5′-UTR of the *crp* mRNA in *Y. pseudotuberculosis* is reported to have two transcriptional start sites that produce a 201 and 287 nt long 5′-UTR each, that are predicted to contain riboswitch-like elements (discussed below). It remains to be resolved whether these are legitimate riboswitches and how they function. Additionally, at 37°C the compromised growth phenotype exhibited by a *Y. pestis hfq* mutant can be partially restored by synthesis of Crp, emphasizing that Hfq and Crp are functionally coordinated during *Y. pestis* infection of its mammalian host (Lathem et al., 2014). Because loss of both Crp or Hfq result in attenuation in virulence for both *Y. pestis* and *Y. pseudotuberculosis*, it appears that subtle differences in their regulatory targets may be important in fine tuning virulence in keeping with host nutritional environment. However, the disparate relationship between Crp and Hfq in *Y. pestis* and *Y. pseudotuberculosis* is likely another aspect of the alterations in their post-transcriptional regulatory cascades that drive their distinct disease manifestations. Importantly the *Y. pestis* Crp regulon incorporates the major virulence factor, Pla, and is involved in its direct transcriptional regulation (Zhan et al., 2008; Lathem et al., 2014).

Several direct targets of Crp have been experimentally verified in both *Y. pseudotuberculosis* (Heroven et al., 2012b) and *Y. pestis* (Zhan et al., 2008) including several sRNAs (Nuss et al., 2015). As sRNAs have vital roles in the regulation of carbon metabolism and nutritional stress adaptation, it makes sense that their regulatory function would be closely aligned with that of Crp. The recent study by Nuss et al. (2015) that generated the first single nucleotide resolution transcriptome profile of *Y. pseudotuberculosis* wild type and *crp* mutant strains, uncovered a complex regulatory network composing transcriptional regulators and 53 sRNAs dysregulated by Crp. Northern blotting confirmed expression of 15 of these sRNAs. Further, a newly identified trans-encoded sRNA (Ysr206), as well as two antisense sRNAs (Ysr232 and Ysr114) exclusively expressed in the *crp* mutant were validated as directly targeted with Crp using gel shift assays. Thus key findings regarding Crp regulation of sRNAs, are that Crp acts as a master regulator of many sRNAs; it binds directly or mediates indirect control of sRNAs through other transcriptional regulators, and that Crp regulation of sRNAs occurs especially at stationary growth phase during catabolite repression.

Earlier work by Yan et al. (2013) demonstrated that 10 out of 104 sRNAs identified in the avirulent *Y. pestis* bv. microtus strain, contained Crp-binding sites. Northern analysis verified expression of seven of these sRNAs in the wild type versus the *crp* mutant. The known Crp-regulated CyaR/RyeE sRNA (Johansen et al., 2008; Papenfort et al., 2008) was amongst those identified. Three novel sRNAs were demonstrated to be directly regulated by Crp: sRNAs sR084 encoded in an IG on the pPCP1 plasmid, as well as sR065, are positively regulated, whereas sR066 seem to be negatively regulated by Crp. Deletion mutants of the CyaR and sR084 sRNAs that are expressed in the lungs were not attenuated as was demonstrated by subcutaneous infection in mice. While expression of CyaR at 26°C is Crp-dependent in *Y. pestis*, it is not the case in *Y. pseudotuberculosis* (Nuss et al., 2015) which may be important for adaptation of *Y. pestis* to the flea environment; and likely represents another difference in sRNA regulation that distinguishes the diseases cause by these two pathogens.

Given that for Crp, there appears to be some distinct differences between Crp-regulated targets and its regulation by Hfq between *Y. pestis* vs. *Y. pseudotuberculosis*, a comparative single nucleotide resolution transcriptomic profile of *Y. pestis* similar to that described for *Y. pseudotuberculosis*, is essential. This could provide a more informed understanding of the varied Crp-dependent sRNAs and their targets. Utilization of this technique should be extended to the various sites of infection (e.g., intestines, spleen, lungs, and lymph nodes) of bacteria during systemic dissemination to identify the localized, nutrient-specific disparate regulatory changes in gene expression of the two pathogens that likely fine tunes their adaptation during infection.

## Post-Transcriptional Regulation Mediated by mRNA Structural Elements

Long 5′-UTRS of some mRNAs can contain structured *cis*-acting non-coding RNA regulatory elements like riboswitches and RNA thermometers. These elements have a distinctive regulatory mechanism in that they undergo structural rearrangement in response to binding of a small ligand, e.g., metabolites or cofactors, or thermal shifts, i.e., their function is intimately coupled with signal integration.

### RNA Thermosensors

Post-transcriptional regulation by RNA thermometers involves the thermally induced unfolding of RNA secondary structures that restricts accessibility to the RBS, and alters translation. Thus far, only one RNA thermometer has been described for the yersiniae. Yet, regulation by such elements seems highly appropriate for *Yersinia* species that exhibit thermo-regulation of gene transcription as the bacteria transitions between the environmental/flea environment into the mammalian host. The thermometer element was found located intercistronically between the *yscW* that encodes a structural component of the T3SS apparatus and the *lcrF* gene that encodes a transcriptional activator of genes encoding effector proteins. The RNA thermometer composes two hairpin mRNA secondary structures. The *lcrF* proximal hairpin contains a sequence of four U’s, also called fourU that sequesters the SD sequence, while the secondary structure occludes the start codon. The mammalian host body temperature is, however, sufficient to melt the unstable G-U bonds that hold the SD/fourU sequences together. Once the hairpin structure is destabilized, post-transcriptional controlled initiation of *lcrF* translation ensues leading to differential synthesis of YscW and LcrF proteins and expression of virulence function. Due to the 100% conservation in nucleotide sequence homology in this region, this RNA thermometer mechanism of regulation is likely conserved in all pathogenic *Yersinia* species. Currently the only clearly defined recognition motifs that typify thermometer elements are the fourU and ROSE elements (Krajewski and Narberhaus, 2014), limiting potential of whole genome based *in silico* prediction of such elements.

### RNA Riboswitches

Unlike RNA thermometer elements, riboswitches require binding of a ligand, usually a metabolite or cofactor, to induce conformational changes in the RNA secondary that directs premature transcription termination or inhibit initiation of translation. A search for riboswitch elements in *Yersinia* species has been neglected until the recent achievement of a high resolution whole genome transcriptomics of *Y. pseudotuberculosis* (Nuss et al., 2015). In this study, 155 mRNAs composed 5′-UTR regions >200 nucleotides in length which make them prime candidates to encode *cis*-acting regulatory elements like riboswitches. A bioinformatic search for riboswitch-like elements (RLEs) using the RibEx riboswitch explorer (Abreu-Goodger and Merino, 2005) predicted four known and an additional 17 RLEs involved in metabolic and gene expression functions, within these long 5′-UTRs. An example of this is that the mRNA of the *btuB* gene that encodes a Ton-B dependent vitamin B12 receptor, composed of a 315 nucleotide long 5′-UTR, contains a known cobalamin riboswitch element. Unknown RLEs require experimental verification, functional characterization and determination of the metabolites/cofactor ligands that mediate their structural switching.

To date a detailed riboregulatory mechanism of a 5′-UTR Mg^2+^ responsive riboswitch, *mgtA*, which regulates the expression of the downstream encoded Mg^2+^ transporter MgtA, has been described only for *Y. enterocolitica* (Korth and Sigel, 2012). Here, in the 5′-UTR, two stem loops structures form at high Mg^2+^ concentration to inhibit expression of MgtA. At low Mg^2+^ concentration an alternate single stem loop antiterminator structure forms that favors translation of the *mgtA* mRNA and Mg^2+^ uptake. Similar studies are required for the *Y. pestis* and *Y. pseudotuberculosis* to identify disparities in riboswitch mechanisms that may facilitate their distinct host infection phenotypes.

## Conclusion

In the last decade, research on sRNA identification and functional analysis has begun to reveal a previously hidden regulatory layer in the already complex gene networks that control cellular function and behavior. As discussed in this review, sRNAs have been shown to act as regulators of *Yersinia* virulence and host adaptation. The underlying emerging theme of the *Yersinia* sRNA studies reviewed here is that sRNAs coordinate metabolic adaptation to enhance the host–pathogen interaction.

Many fundamental questions about sRNA biology remain to be answered, however, before sRNAs can be exploited to disrupt the host–pathogen interaction. For most identified *Yersinia* sRNAs, the exact cellular function and downstream mRNA targets remain to be elucidated. Thus far we have learned that different growth and treatment conditions uncover distinct sRNAs repertoires reflective of that particular set of conditions. So while *in vitro* growth studies representing infection relevant conditions have potential to identify some important host-specific sRNAs, the full complement of these molecules may be overlooked without *in vivo* studies. Only a single study identified *Y. pestis* sRNAs in the biologically relevant context of the lung and spleen, but only one highly induced, and previously annotated sRNA, SsrA, was shown to be important for mouse infection (Yan et al., 2013) similar to a previous study (Okan et al., 2010). Studies identifying, validating and functionally charactering the roles of sRNAs in the various biologically relevant host tissues may prove to be useful in determining the host-specific and niche dependent regulatory mechanisms crucial for virulence of *Yersinia* species. Importantly, comparative analyses of sRNAs and post-transcriptional regulation between *Y. pestis* and *Y. pseudotuberculosis* may provide insight into the evolution of the distinct disease states of these two pathogens.

Because experimental efforts to determine cellular function of sRNAs are time-consuming and labor-intensive, and bioinformatics prediction of target mRNAs remains largely unreliable being confounded by imperfect complementarity between the sRNA and mRNA, researchers are faced with several challenges in the field of sRNA biology. Undoubtedly, however, the mechanisms of sRNA regulation suggests the possibility that inhibition of key sRNA folding or targeted mRNA interactions strategies can be developed as the basis of novel anti-infective strategies, especially in the face of antibiotic resistance. The several instances of obviously different post-transcriptional regulatory control of infection and adaptation between *Y. pestis* and *Y. pseudotuberculosis* discussed in this review, emphasize their important and specific roles in fine tuning adaptation of these pathogens to cause disease.

## Author Contributions

LM-C and VV reviewed the literature and contributed to writing and revising this manuscript.

## Conflict of Interest Statement

The authors declare that the research was conducted in the absence of any commercial or financial relationships that could be construed as a potential conflict of interest.
