# Supplementary material for: Yersinia pestis and Yersinia pseudotuberculosis infection: a regulatory RNA perspective
Source: Front Microbiol. 2015 Sep 17;6:956. doi: 10.3389/fmicb.2015.00956 (PMC4585118; doi:10.3389/fmicb.2015.00956)
Supplement: Supplementary file 1 [file Table_1.DOC]

**Table S1. Confirmed/Validated sRNAs in *Yersinia pestis* and *Yersinia pseudotuberculosis***

|  | **Strain** | | **Temperature** | | **Validation** | | **Hfq-dependence** | |  |  |
| --- | --- | --- | --- | --- | --- | --- | --- | --- | --- | --- |
| **sRNA** | **Yptb** | **Yp** | **26-28^o^C** | **37^o^C** | **N/RT** | **R** | **26-28^o^C** | **37^o^C** | **Growth phase-condition induction^h^ Regulation/infection data** | **Reference** |
|  |  |  |  |  |  |  |  |  |  |  |
| **Ysr1** | x | x | No | x | N |  | ND | ND | S (37^o^C, Yptb) | Koo 2011, Schiano 2014 |
| **Ysr2** | x | x | x | x | N |  | ND | ND | EL, ML (37^o^C, Yptb); S (26^o^C, Yptb) | Koo 2011, Schiano 2014 |
| **Ysr3** | x | x | x | x | N |  | ND | ND | LL(26^o^C, Yptb); ML (37^o^C, Yptb) | Koo 2011, Schiano 2014 |
| **Ysr4** | x | x | Low | Low | N | x | Yptb(+) | Ypt(+), Yp(+) | S Yptb (26^o^C and 37^o^C); LL, Yp (37^o^C) | Koo 2011, Schiano 2014 |
| **Ysr5** | x | x | Low | Low | N |  | ND | ND | EL (37^o^C, Yptb); S (26^o^C Yptb) | Koo 2011, Schiano 2014 |
| **Ysr6** | x | x | Low | Low | N |  | ND | ND | ML (37^o^C, Yptb); S (26^o^C, Yptb) | Koo 2011, Schiano 2014 |
| **Ysr7/154/MicA/sr004** | x | x | x | x | N | x | Yp (+) | Yp (+), Yptb (+) | S (Yptb, 26^o^C); LL,S (Yp, 37^o^C); EL, ML (Yptb, 37^o^C) | Koo 2011, Beauregard 2013, Schiano 2014, Yan 2013 |
| **Ysr9** | x | x | Low | x | N |  | ND | ND | ML (Yptb, 37^o^C); S (Yptb, 37^o^C) | Koo 2011, Schiano 2014 |
| **Ysr10** | x | x | Low | x | N |  | ND | ND | ML (Yptb, 37^o^C); S (26^o^C, Yptb) | Koo 2011, Schiano 2014 |
| **Ysr11/166/FnrS** | x | x | x | x | N | x | Yptb (-) | Yptb(+), Yp(+) | LL (Yptb, 37^o^C), S (Yptb, 26^o^C); LL (Yp, 26^o^C); ML, LL (Yp, 37^o^C) | Koo 2011, Beauregard 2013, Schiano 2014 |
| **Ysr12** | x | x | Low | x | N |  | ND | ND | EL, ML (37^o^C, Yptb); S (26^o^C, Yptb) | Koo 2011, Schiano 2014 |
| **Ysr15** | x | x | Low | x | N |  | ND | ND | ML, S (37^o^C, Yptb); S (Yptb, 37^o^C) | Koo 2011, Schiano 2014 |

| **Ysr16** | x | x | No | x | N |  | ND | ND | ML (37^o^C, Yptb) | Koo 2011, Schiano 2014 |
| --- | --- | --- | --- | --- | --- | --- | --- | --- | --- | --- |
| **Ysr17/sR051** | x | x | x | x | N |  | ND | ND | EL, ML (37^o^C, Yptb); S (26^o^C, Yptb) | Koo 2011, Yan 2013, Schiano 2014 |
| **Ysr18** | x | x | x | x | N |  | ND | ND | EL, ML (37^o^C, Yptb); S (26^o^C, Yptb) | Koo 2011, Schiano 2014 |
| **Ysr19** | x | x | Low | Low | N |  | ND | ND | S (37^o^C, 26^o^C, Yptb) | Koo 2011, Schiano 2014 |
| **Ysr20** | x | x | x | x | N |  | No | Yptb(+), Yp(+) | LL, S (26^o^C Yptb); ML-S (37^o^C, Yptb); LL (26^o^C, Yp); ML, LL (26^o^C Yp) | Koo 2011, Schiano 2014 |
| **Ysr22** | x | x | Low | Low | N |  | ND | ND | LL (37^o^C, Yptb); S (26^o^C, Yp) | Koo 2011, Schiano 2014 |
| **Ysr23/160** | x | x | x | x | N | x | Yptb (-) | Yptb (-), Yp(+) | LL, S (26^o^C, 37^o^C, Yptb); LL (26^o^C, Yp); EL-LL (37^o^C, Yp) Not attenuated in mice IG (Yptb) and IN (Yp) | Koo 2011, Beauregard 2013, Schiano 2014 |
| **Ysr24** | x | x | No | x | N |  | ND | ND | ML (37^o^C, Yptb) | Koo 2011, Schiano 2014 |
| **Ysr25** | x | x | x | x | N |  | ND | ND | ML (37^o^C, Yptb); S (26^o^C, Yptb) | Koo 2011, Schiano 2014 |
| **Ysr26** | x | x | No | x | N |  | ND | ND | ML (37^o^C, Yptb) | Koo 2011, Schiano 2014 |
| **Ysr29** | x^a^ | No | Low | x | N | x | ND | ND | S (26^o^C, Yptb); ML (37^o^C, Yptb) Attenuated in mice IG (Yptb) | Koo 2011 |
| **Ysr34** | x | x | No | Low | N |  | ND | ND | LL (37^o^C, Yptb) | Koo 2011, Schiano 2014 |
| **Ysr35** | x | x | x | x |  | x | Yptb | ND | ML (37^o^C, Yptb); S (26^o^C, Yptb) Attenuated in mice IG (Yptb) and IN (Yp) | Koo 2011, Schiano 2014 |
| **Ysr36** | x | x | Low | Low | N |  | ND | ND | C (37^o^C, Yptb); S (26^o^C, Yptb) | Koo 2011, Schiano 2014 |
| **Ysr45/180/GcvB/6S RNA/ sR013** | x | x | x | x | N | x | No | Yp (+), Yptb (+) | S (26^o^C Yptb); EL (37^o^C Yptb); LL (26^o^C, Yp); ML, LL (37^o^C, Yp) | Beauregard 2013, Koo 2011, Yan 2013, Schiano 2014 |
| **Ysr46** | x | x | Low | x | N |  | ND | ND | EL (26^o^C, 37^o^C, Yptb) | Koo 2011, Schiano 2014 |

| **Ysr48/RybB/ sR023** | x | x | x | x | N/RT | x | Yp(+) | Yptb(+), Yp(+) | S (26^o^C, 37^o^C, Yptb); S (26oC, Yp); ML (37^o^C, Yp); UR in infected lungs Not attenuated in mice IG (Yptb) and IN (Yp) | Koo 2011, Yan 2013, Schiano 2014 |
| --- | --- | --- | --- | --- | --- | --- | --- | --- | --- | --- |
| **Ysr65/175** | x | x | x | x | N | x | Yp (+) | No | ND | Beauregard 2013, Koo 2011, Schiano 2014 |
| **Ysr73/169** | x | x | x | x | N | x | No | Yp (-) | ND | Beauregard 2013, Koo 2011, Schiano 2014 |
| **Ysr88/152** | x | x | Yp | x | N | x | Yptb (-) | slighty Yp and Yptb | ND | Beauregard 2013, Koo 2013, Schiano 2014 |
| **Ysr141 (pCD1)** | x | x | x | x | N | x | ND | ND | ML, LL (37^o^C, Yp) Regulates T3SS | Schiano 2014, Koo 2011 |
| **Ysr142 (pCP1)** | No | x | x | x | N | x | ND | ND | S (37^o^C, Yp) | Schiano 2014 |
| **Ysr143** | No | x | Low | x | N |  | ND | ND | C (37^o^C, Yp) | Schiano 2014 |
| **Ysr144 (pMT1)** | No | x | Low | x | N | x | ND | ND | LL (37^o^C, Yp) | Schiano 2014 |
| **Ysr145/157** | x | x | x | x | N | x | Yp (+), Yptb (+) | Yp (+), Yptb (+) | ND | Beauregard 2013, Koo 2011, Schiano 2014 |
| **Ysr146.2/187/ sR024** | x | x | Yp | x | N/RT | x | No | Yptb (-); Yp (+)^h^ | UR in infected lungs CRP-independent | Koo 2011, Beauregard 2013, Schiano 2014, Yan 2013 |
| **Ysr148/153/ GlmZ/ sR016** | x | x | x | x | N | x | Yp (+) | Yp (+) | ND | Koo 2011, Beauregard 2013, Schiano 2014, Yan 2013 |
| **Ysr149/181/ sR014** | x | x | x | x | N | x | Yp (+), Yptb (+) | Yp (+), Yptb (+) | ML, S (37^o^C, Yp) | Koo 2011, Beauregard 2013, Schiano 2014, Yan 2013 |
| **Ysr151/RnpB/ sR007** | x | x | x | x | N |  | Yp (+) | slighty Yp (+) | ND | Beauregard 2013, Yan 2013 |

| **Ysr155/RyfD/ Yp-sR6** | x | x | x | x | N | x | Yp (+), Yptb (-) | No | ND | Beauregard 2013, Qu 2012 |
| --- | --- | --- | --- | --- | --- | --- | --- | --- | --- | --- |
| **Ysr156/Ffs/ Yp-sR29/ sR020/ 4.5S RNA** | x | x | x | x | N/RT | x | No | No | S (26^o^C, Yp); Id, LMg (37^o^C, Yp); DR in infected lungs | Beauregard 2013, Qu 2012, Yan 2013 |
| **Ysr158** | x | x | x | x | N | x | Yp (+), Yptb (+) | Yp (+), Yptb (+) | ND | Beauregard 2013 |
| **Ysr159/cyaR/ Yp-sR30/ sR012/RyeE** | x | x | x | x | N/RT | x | Yp (+), Yptb (+) | Yp (+), Yptb (+) | Id, Lca (37^o^C, Yp); C (26^o^C Yp); UR in infected lungs and nutrient limitation (Yp) CRP-regulated (+); not attenuated in mice SC/IN | Beauregard 2013, Qu 2012, Yan 2013 |
| **Ysr161** | x | x | x | x | N | x | Yptb(+) | Yp (+), Yptb (+) | ND | Beauregard 2013 |
| **Ysr163** | x | x | x | x | N |  | No | Yp (-) | ND | Beauregard 2013 |
| **Ysr164** | x | x | x | x | N | x | Yp (+), Yptb (+) | Yp (+), Yptb (+) | ND | Beauregard 2013, Schiano 2014 |
| **Ysr165/Yp-sR38** | x | x | x | x | N/RT | x | Yp (+), Yptb (-) | No | Id (37^o^C, Yp) | Beauregard 2013, Qu 2012 |
| **Ysr167** | x | x | x | xx | N |  | No | No | ND | Beauregard 2013 |
| **Ysr170** | x | x | x | xx | N | x | Yp(+) | Yp (+)b | ND | Beauregard 2013 |
| **Ysr171** | x | x | x | xx | N | x | No | Yp (+), Yptb (+) | ND | Beauregard 2013 |
| **Ysr172** | x | x | Yp | Yp | N | x | Yp (+)d | Yp (+)d | ML, LL, S (37^o^C, Yp) | Beauregard 2013, Schiano 2014 |
| **Ysr174** | x | x | No | xx | N | x | No | Yptb (+) | ND | Beauregard 2013 |

| **Ysr177** | x | x | x | Yptb | N | x | Yp (+) | Yptb (-) | ND | Beauregard 2013 |
| --- | --- | --- | --- | --- | --- | --- | --- | --- | --- | --- |
| **Ysr179/CsrB/ sR003** | x | x | x | x | N/RT |  | Yptb (-), Yp (-)^d^ | Yptb (+) Yp(+) | DR in infected lungs | Beauregard 2013, Yan 2013 |
| **Ysr182/6S RNA SsrS/Yp-sR28/ Yp-sR1/sR017** | x | x | x | x | N/RT | x | Yptb(+) | No | S (26^o^C, Yp); UR in infected lungs Not attenuated SC in mice | Beauregard 2013, Qu 2012, Yan 2013 |
| **Ysr183/SroG** | x | x | x | x | N | x | No | Yptb (+) | ND | Beauregard 2013 |
| **Ysr185/CsrC/ sR026/Ysr188** | x | x | x | x | N/RT | x | Yp (+) | Yp (+), Yptb (+) | DR in infected lungs | Beauregard 2013, Yan 2013, Schiano 2014 |
| **Ysr186/Spot42** | x | x | x | x | N | x | Yp (+) | Yptb (-) | ND | Beauregard 2013 |
| **Ysr199** | No | x | x | x | N | x | ND | ND | C but very low (37^o^C Yp) | Schiano 2014 |
| **Ysr218** | x | x | x | x | N |  | ND | ND | C, not in S (37^o^C Yp) | Schiano 2014 |
| **Ysr230** | x | x | x | x | N |  | ND | ND | EL (37^o^C, Yp) | Schiano 2014 |
| **Ysr249** | x | x | x | x | N |  | ND | ND | EL (37^o^C, Yp) | Schiano 2014 |
| **Yp-sR2** | x | x | x | x | N/RT |  | ND | ND | S (26^o^C, Yp) | Qu 2012 |
| **Yp-sR3** | x | x | x | x | N/RT |  | ND | ND | S (26^o^C, Yp) | Qu 2012 |
| **Yp-sR4** | No | x | x | x | RT |  | ND | ND | S (26^o^C, Yp) | Qu 2012 |
| **Yp-sR7** | x | x | x | x | N/RT |  | ND | ND | Not in S at 26^o^C (Yp) | Qu 2012 |
| **Yp-sR8** | x | x | x | x | N/RT |  | ND | ND | S (26^o^C, Yp) | Qu 2012 |
| **Yp-sR9** | x | x | x | x | RT |  | ND | ND | S (26^o^C, Yp) | Qu 2012 |
| **Yp-sR10** | x | x | x | x | RT |  | ND | ND | S (26^o^C, Yp) | Qu 2012 |
| **Yp-sR13** | x | xc | x | x | RT |  | ND | ND | S (26^o^C, Yp) | Qu 2012 |
| **Yp-sR14** | x | x | x | x | RT |  | ND | ND | S (26^o^C, Yp) | Qu 2012 |
| **Yp-sR15** | x | x | x | x | RT |  | ND | ND | S (26^o^C, Yp) | Qu 2012 |
| **Yp-sR16** | x | x | x | x | N |  | ND | ND | LMg, LCa (37^o^C, Yp) | Qu 2012 |
| **Yp-sR17** | x | x | x | x | RT |  | ND | ND | S (26^o^C, Yp); LMg, LCa (37^o^C, Yp) | Qu 2012 |
| **Yp-sR18** | x | x | x | x | RT |  | ND | ND | S (26^o^C, Yp) | Qu 2012 |
| **Yp-sR19** | x | x | x | x | RT |  | ND | ND | S (26^o^C, Yp) | Qu 2012 |
| **Yp-sR20** | x | x | x | x | RT |  | ND | ND | S (26^o^C, Yp) | Qu 2012 |

| **Yp-sR22** | x | x | x | x | RT |  | ND | ND | S (26^o^C, Yp) | Qu 2012 |
| --- | --- | --- | --- | --- | --- | --- | --- | --- | --- | --- |
| **Yp-sR23** | x | x^c^ | x | x | RT |  | ND | ND | S (26^o^C, Yp) | Qu 2012 |
| **Yp-sR25** | x^e^ | x^f^ | x | x | RT |  | ND | ND | S (26^o^C, Yp); Id, LMg (37^o^C, Yp) | Qu 2012 |
| **Yp-sR26** | NR | x | x | x | N/RT |  | ND | ND | S (26^o^C); LMg (37^o^C) | Qu 2012 |
| **Yp-sR27** | NR | x | x | x | RT |  | ND | ND | S (26^o^C, Yp); Id, LCa (37^o^C, Yp) | Qu 2012 |
| **Yp-sR31/sR022** | x | x | x | x | RT |  | ND | ND | LMg, LCa, Id (37^o^C, Yp) | Qu 2012, Yan 2013 |
| **Yp-sR32** | x | x | x | x | N/RT |  | ND | ND | S (26^o^C, Yp) | Qu 2012 |
| **Yp-sR34** | x | x | x | x | RT |  | ND | ND | Id, LCa (37^o^C, Yp) | Qu 2012 |
| **Yp-sR37** | x | x | x | x | N/RT |  | ND | ND | S (26^o^C, Yp); LMg (37^o^C, Yp) | Qu 2012 |
| **Yp-sR43** | x | x | x | x | RT |  | ND | ND | S (26^o^C, Yp) | Qu 2012 |
| **sR009/RyhB2/ Ysr146.1** | x | x | x | x | RT |  | ND | ND | UR in infected lungs Not attenuated SC/IN in mice | Yan 2013, Koo 2011, Schiano 2014 |
| **sR022/tmRNA/ ssrA/Yp-sR31** | x | x |  |  | RT |  | ND | ND | Induced *in vivo* Attenuated SC in mice | Yan 2013, Qu 2012 |
| **sR025 (pPCP1)** | No | x |  |  |  |  | ND | ND | ND | Yan 2013 |
| **sR027** | x | x |  |  | N/RT |  | ND | ND | DR in infected lungs | Yan 2013 |
| **sR034/Ysr59** | x | x | x | Low | N |  | Yp (+)^g^ | | C (26^o^C, Yp) Not attenuated SC/IN in mice | Yan 2013, Koo 2011, Schiano 2014 |
| **sR035/Ysr104** | x | x | x | Low | N |  | ND | ND | S (26^o^C, Yp) Not attenuated SC/IN in mice; regulates biofilm | Yan 2013, Koo 2011, Schiano 2014, Fang 2014 |
| **sR039** | x | x |  |  | RT |  | ND | ND | UR in infected lungs | Yan 2013 |
| **sR043** | x | x |  |  | N |  | Yp (+)^g^ | | ND | Yan 2013 |
| **sR053/Ysr39** | x | x | x | x | N |  | Yp (+)^g^ | | ND | Yan 2013, Koo 2011, Schiano 2014 |
| **sR055** | x | x |  |  |  |  | ND | ND | Not attenuated SC/IN in mice | Yan 2013 |

| **sR065/Ysr110** | x | x | x | x | N |  | ND | ND | CRP-regulated (+) | Yan 2013, Koo 2011, Schiano 2014 |
| --- | --- | --- | --- | --- | --- | --- | --- | --- | --- | --- |
| **sR066** | x | x |  |  | N |  | ND | ND | CRP-regulated (-) | Yan 2013 |
| **sR073** | x | x | x | x | N |  | Yp (+)^g^ | | C (26^o^C, Yp) Not attenuated SC/IN in mice | Yan 2013 |
| **sR076** | x | x |  |  | N |  | ND | ND | CRP-independent | Yan 2013 |
| **sR081 (pCD1)** | x | x |  |  | N |  | Yp (+)^g^ | | ND | Yan 2013 |
| **sR084 (pPCP1)** | No | x | x | x | N |  | Yp (+)^g^ | | S (26^o^C, Yp); UR in oxidative stress CRP-regulated (+), not attenuated SC/IN in mice | Yan 2013 |
| **sR088** | x | x | x | Low | N |  | Yp (+)^g^ | | C (26^o^C, Yp) Not attenuated SC/IN in mice | Yan 2013 |
| **sR100** | x | x |  |  | N |  | Yp (+)^g^ | | ND | Yan 2013 |
| **sR101** | x | x |  |  | N |  | ND | ND | ND | Yan 2013 |
| **sR104 (pPCP1)** | No | x |  |  | N |  | ND | ND | ND | Yan 2013 |

**Yp:** *Y. pestis*; **Yptb:** *Y. pseudotuberculosis*; **N:** Northern blot; **RT:** Real time PCR; **R:** 5’/3’ Race; **ND:** Not determined in *Yersinia* yet; **(+):** positively regulated; **(-):** negatively regulated; **a:** specific to IP32953; **b:** less dependent in CO92 than in KIM; **c:** not in all *Y. pestis* strains; **d:** only in KIM, not in CO92; **e:** not in all *Y. pseudotuberculosis* strains; **f:** not in *Y. pestis* KIM; **g:** temperature not known as a pool of RNA from bacteria grown at 26°C and 37°C was used; **h:** underlined data are based on numbers of deep sequencing; **LL:** late log; **EL:** early log; **ML:** midlog; **S:** stationary; **C:** constant; **LMg:** low Mg; **LCa:** Low calcium; **Id:** iron deprivation; **IG:** intragastric; **SC:** subcutaneously; **IN:** intranasal; **UR:** upregulated; **DR:** downregulated. Those sRNAs encoded on plasmids are underlined and the plasmid on which they are located is indicated within parenthesis
