# Supplementary material for: Yersinia pestis and Yersinia pseudotuberculosis infection: a regulatory RNA perspective
Source: Front Microbiol. 2015 Sep 17;6:956. doi: 10.3389/fmicb.2015.00956 (PMC4585118; doi:10.3389/fmicb.2015.00956)
Supplement: Supplementary file 2 [file Table_2.DOCX]

**Table S2. Comparison of Yersinia pestis sRNAs identified in four separate studies**

| ***Common sRNAs found between studies*** | | | | |
| --- | --- | --- | --- | --- |
| **2 common sRNAs between "Qu 2012" and "Beauregard 2013":** | | |  |  |
| Ysr155/RyfD/Yp-sR6 |  |  |  |  |
| Ysr165/Yp-sR038 |  |  |  |  |
| **3 common sRNAs between "Qu 2012" and "Yan 2013":** | | | | |
| sR099/Yp-sR5 |  |  |  |  |
| sR022/Yp-sR31 |  |  |  |  |
| sR001/Yp-sR33 |  |  |  |  |
| **3 common sRNAs between "Qu 2012", "Beauregard 2013" and "Yan 2013":** | | | | |
| Ysr182/6S RNA/ sR017/Yp-sR28 | |  |  |  |
| Ysr156/Ffs/sR020/Yp-sR29 | |  |  |  |
| Ysr159/CyaR/sR012/Yp-sR30 | |  |  |  |
| **2 common sRNAs between "Beauregard 2013" and "Yan 2013":** | | | | |
| sR007/Ysr151/RnpB |  |  |  |  |
| sR003/Ysr179/CsrB |  |  |  |  |
| **8 common sRNAs between "Beauregard 2013" and "Schiano 2014":** | | | | |
| Ysr88/152 | Ysr11/166/FnrS |  |  |  |
| Ysr145/157 | Ysr73/169 |  |  |  |
| Ysr23/160 | Ysr172 |  |  |  |
| Ysr164 | Ysr65/175 |  |  |  |
| **6 common sRNAs between "Beauregard 2013", "Yan 2013" and "Schiano 2014":** | | | | |
| sR016/Ysr148/153/GlmZ | sR014/Ysr149/181 |  |  |  |
| Ysr7/154/MicA | sR026/Ysr188/Ysr185/CsrC |  |  |  |
| sR013/Ysr45/180/GcvB | sR024/Ysr146.2/187 |  |  |  |
| **1 common sRNA between "Qu 2012", "Yan 2013" and "Schiano 2014":** | | | | |
| Ysr27/sR060/Yp-sR12 |  |  |  |  |
| **16 common sRNAs between "Yan 2013" and "Schiano 2014":** | | |  |  |
| sR005/Ysr150 | sR023/Ysr48 | sR040/Ysr135 | sR059/Ysr229 |  |
| sR009/Ysr146.1 | sR030/Ysr51 | sR049/Ysr222 | sR054/Ysr110 |  |
| sR011/Ysr147 | sR034/Ysr59 | sR051/Ysr17 | sR074/Ysr68 |  |
| sR021/Ysr112 | sR035/Ysr104 | sR053/Ysr39 | sR092/Ysr92 |  |
| **0 common sRNAs between "Qu 2012", "Beauregard 2013", "Yan 2013" and "Schiano 2014":** | | | | |
| **0 common sRNAs between "Qu 2012" and "Schiano 2014":** | | |  |  |
| **0 common sRNAs between "Qu 2012", "Beauregard 2013" and "Schiano 2014":** | | |  |  |
|  |  |  |  |  |
|  |  |  |  |  |
| ***Exclusive sRNAs found in each study*** | | | | |
| **34 sRNAs found exclusively in "Qu et al 2012":** | | | | |
| Yp-sR1 | Yp-sR13 | Yp-sR22 | Yp-sR36 |  |
| Yp-sR2 | Yp-sR14 | Yp-sR23 | Yp-sR37 |  |
| Yp-sR3 | Yp-sR15 | Yp-sR24 | Yp-sR39 |  |
| Yp-sR4 | Yp-sR16 | Yp-sR25 | Yp-sR40 |  |
| Yp-sR7 | Yp-sR17 | Yp-sR26 | Yp-sR41 |  |
| Yp-sR8 | Yp-sR18 | Yp-sR27 | Yp-sR42 |  |
| Yp-sR9 | Yp-sR19 | Yp-sR32 | Yp-sR43 |  |
|  | | | | |
|  | | | | |
| **Table 1 continued** | | | | |
| **34 sRNAs found exclusively in "Qu et al 2012" continued:** | | | | |
| Yp-sR10 | Yp-sR20 | Yp-sR34 |  |  |
| Yp-sR11 | Yp-sR21 | Yp-sR35 |  |  |
| **10 RNAs found exclusively in "Beauregard 2013":** | | | | |
| Ysr158 | Ysr173 (rmf) |  |  |  |
| Ysr161 | Ysr174 |  |  |  |
| Ysr163 | Ysr177 |  |  |  |
| Ysr167 | Ysr183 (SroG) |  |  |  |
| Ysr170 | Ysr186 (Spot42) |  |  |  |
| **73 RNAs found exclusively in "Yan 2013":** | | | | |
| sR002 | sR041 | sR067 | sR087 |  |
| sR006 | sR042 | sR068 | sR088 |  |
| sR008 | sR043 | sR069 | sR089 |  |
| sR010 | sR044 | sR070 | sR090 |  |
| sR015 | sR045 | sR071 | sR091 |  |
| sR018 | sR046 | sR072 | sR093 |  |
| sR019 | sR047 | sR073 | sR094 |  |
| sR022 | sR048 | sR075 | sR095 |  |
| **179 RNAs found exclusively in "Schiano 2014" (these numbers are prefixed by a Ysr):** | | | | |
| 1 | 46 | 95 | 138 | 220 |
| 2 | 47 | 96 | 139 | 221 |
| 3 | 49 | 97 | 140 | 223 |
| 4 | 50 | 98 | 141 | 224 |
| 5 | 52 | 99 | 142 | 225 |
| 6 | 54 | 100 | 143 | 226 |
| 8 | 55 | 101 | 144 | 227 |
| 9 | 56 | 102 | 189 | 228 |
| 10 | 57 | 103 | 190 | 230 |
| 12 | 58 | 105 | 191 | 231 |
| 13 | 60 | 106 | 192 | 232 |
| 14 | 61 | 107 | 193 | 233 |
| 15 | 62 | 108 | 194 | 234 |
| 16 | 63 | 109 | 195 | 235 |
| 18 | 64 | 111 | 197 | 236 |
| 19 | 66 | 113 | 198 | 237 |
| 20 | 67 | 114 | 199 | 238 |
| 21 | 69 | 116 | 200 | 239 |
| 22 | 71 | 117 | 201 | 240 |
| 24 | 72 | 119 | 202 | 241 |
| 25 | 74 | 120 | 203 | 242 |
| 26 | 75 | 121 | 204 | 243 |
| 28 | 76 | 122 | 205 | 244 |
| 30 | 77 | 123 | 206 | 245 |
| 31 | 78 | 124 | 207 | 246 |
| 32 | 79 | 125 | 208 | 247 |
| 33 | 80 | 126 | 209 | 248 |
| 34 | 81 | 127 | 210 | 249 |
| 35 | 82 | 128 | 211 | 250 |
| 36 | 83 | 129 | 212 | 251 |
|  |  |  |  |  |
| **Table 1 continued** |  |  |  |  |
| **179 RNAs found exclusively in "Schiano 2014" (these numbers are prefixed by a Ysr) continued:** | | | | |
| 37 | 85 | 130 | 213 |  |
| 38 | 86 | 131 | 214 |  |
| 40 | 87 | 132 | 215 |  |
| 41 | 89 | 133 | 216 |  |
| 42 | 90 | 134 | 217 |  |
| 43 | 91 | 136 | 218 |  |
| 44 | 93 | 137 | 219 |  |

([Qu et al., 2012](#_ENREF_80);[Beauregard et al., 2013](#_ENREF_8);[Yan et al., 2013](#_ENREF_111);[Schiano et al., 2014](#_ENREF_87))
